# Elements of Long-Term Care That Promote Quality of Life for Indigenous and First Nations Peoples: A Mixed Methods Systematic Review

**DOI:** 10.1093/geront/gnac153

**Published:** 2022-10-14

**Authors:** Deborah Brooks, Sandra Johnston, Christina Parker, Leonie Cox, Melissa Brodie, Catherine Radbourne, Margaret MacAndrew

**Affiliations:** School of Nursing, Queensland University of Technology, Brisbane, Queensland, Australia; School of Nursing, Queensland University of Technology, Brisbane, Queensland, Australia; School of Nursing, Queensland University of Technology, Brisbane, Queensland, Australia; School of Nursing, Queensland University of Technology, Brisbane, Queensland, Australia; School of Nursing, Queensland University of Technology, Brisbane, Queensland, Australia; Library Services, Queensland University of Technology, Brisbane, Queensland, Australia; School of Nursing, Queensland University of Technology, Brisbane, Queensland, Australia

**Keywords:** First Nations peoples, Indigenous Elders, Nursing homes, Older people, Residential aged care

## Abstract

**Background and Objectives:**

Little is known about elements of long-term care (LTC) that promote quality of life (QoL) for older Indigenous and First Nations peoples. This systematic review aimed to extend understanding of those deemed most important.

**Research Design and Methods:**

Following Preferred Reporting Items for Systematic Reviews and Meta-Analyses (PRISMA) guidelines, systematic database and hand-searching were used to find published and unpublished qualitative studies and textual reports. A convergent integrated approach was used to synthesize data, according to the Joanna Briggs Institute methodology for mixed methods systematic reviews.

**Results:**

Included papers (11 qualitative; seven reports) explored views and experiences of Indigenous residents, families, and LTC staff from North America (8), South Africa (1), Norway (1), New Zealand (1), and Australia (7). Elements of care included: (a) codesigning and collaborating with Indigenous and First Nations communities and organizations to promote culturally safe care; (b) embedding trauma-informed care policies and practices, and staff training to deliver culturally safe services; (c) being respectful of individual needs, and upholding cultural, spiritual and religious beliefs, traditional activities and practices; (d) promoting connection to culture and sense of belonging through sustained connection with family, kin, and Indigenous and First Nations communities.

**Discussion and Implications:**

This review identifies elements or models of care that promote QoL for Indigenous and First Nations peoples in LTC. While included papers were mostly from the United States and Australia, the congruence of elements promoting QoL was evident across all population groups. Findings may be used to inform standards specific to the care of Indigenous and First Nations peoples.

**Implications for Practice**

1. Culturally appropriate care for one group of people or community may not be the case elsewhere, so services are best developed locally; the codesign of services in collaboration with Indigenous and First Nations communities and organizations honors the cultural safety principles of power sharing and working in partnership.
2. Strengthening the Indigenous and First Nations workforce by enhancing their recruitment and retention and building the confidence and capacity of non-Indigenous health workers through cultural safety education and practice to work kindly and respectfully with Indigenous and First Nations peoples, are priorities.
3. It is imperative that trauma-informed care is embedded in aged care services as a priority due to the significantly higher burden of trauma, chronic disease, and other markers of disadvantage that are a result of centuries of ongoing colonization of Indigenous and First Nations peoples.

## Background and Objectives

As the number of older Indigenous and First Nations peoples increases, so does the need for health and social care services, including long-term care (LTC). There is no agreed definition of the term “Indigenous Peoples”; however international organizations refer to self-identification as belonging to an Indigenous group or culture; connection to geographically distinct ancestral territories; descent from peoples who lived in such areas before colonization; and maintenance of distinct cultural, linguistic, and social identities (World Health Organization [WHO], 2003). The terms “First Peoples” and “First Nations” are more widely used in Australian and Canadian literature to describe the many and varied peoples affected by colonization (Deravin et al., 2021; Peters & Mika, 2017). While the use of such generic terms is contentious (Peters & Mika, 2017), they are frequently used within the research and policy literature (Flanagin et al., 2021; Peters & Mika, 2017; United Nations, 2009). An estimated 370 million Indigenous peoples live in 70 countries across the world (United Nations, 2009). These peoples are more likely to experience ill-health and have a lower life expectancy than non-Indigenous people living in the same country (United Nations, 2016); a direct consequence of structural inequalities and discrimination resulting from colonization, marginalization, and intergenerational trauma (Menzies, 2019). As such, they may require home care services and residential (“aged”) care at a younger age than non-Indigenous peoples (e.g., age 50 compared to age 65 in Australia; Australia Government, 2017). They may also experience greater barriers in accessing culturally safe and appropriate services (Keelan et al., 2021; United Nations, 2016), including LTC settings such as nursing or residential care homes, which can reduce quality of life (QoL).

While widely acknowledged that “quality of life is an imprecise concept that is difficult to define,” there is consensus that good QoL should be an essential outcome of LTC (Courtney, 2003, p. 58), with consumer satisfaction often being used as a proxy measure (Schalock, 2004). QoL in LTC settings may encompass: comfort, functional competence, autonomy, dignity, privacy, individuality, meaningful activity, relationships, enjoyment, security, and spiritual well-being (Kane et al., 2003). Specific factors identified as integral to QoL for older Indigenous Australians include family and friends, spending time on Country, connection to the Aboriginal community, connection to culture, health, respect, Eldership, family support and culturally safe and appropriate services, safety and security, spirituality, future plans (including end of life), and meeting basic needs (Smith et al., 2020). Based on these key factors, the “Good Spirit, Good Life” framework has been used to guide understanding of the QoL within Indigenous Australians, however, it has not been applied to other Indigenous populations worldwide (Smith et al., 2020). A recent review of dimensions of quality at end of life, identified the life and death journey, a belief in spirits, tribally grounded traditions, dominant cultural religion influences, a family focus, optimization of holistic health, communication and access to appropriate resources, and end-of-life death rituals, as being important to Indigenous peoples, particularly within a North American context (Terpstra et al., 2021).

Cultural safety and Trauma and Violence Informed Care are central to improving health outcomes for Indigenous Australians (Australia Government, 2013; Horrill, 2020), and Indigenous and First Nations peoples in many contexts internationally who were subject to removal from families/communities and incarceration in institutions; a process creating those known as “the stolen generations” (Read, 1982). The ongoing intergenerational trauma of these events cannot be overstated: “The bereavement experienced by many forcibly removed Indigenous children was traumatic” (Human Rights and Equal Opportunity Commission, 1997, p. 161). It is a mistake to see these events as “past” as they are not only in the living memory of older Indigenous and First Nations peoples, but the traumas extend into the present time and manifest in multiple ways on people’s health and well-being and on engagement with service providers. Experiences of discrimination, racism, and institutional abuse may therefore lead to hesitancy and lack of engagement of Indigenous and First Nations peoples with LTC (Royal Commission into Aged Care Quality and Safety, 2021, pp. 73–74).

Respecting human rights and meeting the unique needs of older Indigenous and First Nations peoples residing in LTC holistically is, therefore, an important aspect of care. In doing so racism, intersections of discrimination such as age and gender, and complex grief and trauma are addressed. There is a need to develop “effective models of care and best practices so that such programs and services are culturally and linguistically appropriate for Indigenous peoples” (United Nations, 2016, p. 3) and are culturally safe (Davy et al., 2016). This review aimed to extend our understanding of models of care (a framework or structured approach to providing care to a specific population [Luckett et al., 2014]) that promotes QoL for Indigenous and First Nations peoples living in LTC by identifying, appraising, and synthesizing relevant literature. A preliminary search in PROSPERO, the Joanna Briggs Institute (JBI) Database, and the Cochrane Library of Systematic Reviews found no existing systematic reviews on this topic (October 2020). This review was conducted according to a priori protocol (PROSPERO CRD42020218542). The review question was: Which elements or models of care promote QoL or consumer satisfaction for Indigenous and First Nations peoples living in LTC?

## Method

This was a mixed methods systematic review following the Preferred Reporting Items for Systematic Reviews and Meta-Analyses (PRISMA) reporting guidelines (Page et al., 2021), the JBI methods for mixed methods systematic reviews (Aromataris, 2020; Lizarondo et al., 2020) and the ConQual approach to assessing confidence in qualitative evidence synthesized findings (Munn et al., 2014).

### Eligibility Criteria

#### Types of participants

Indigenous and First Nations peoples living in LTC communities/facilities. We (the authors) acknowledge that the use of collective nouns to describe Indigenous and First Nations Peoples is problematic (Peters & Mika, 2017). However, based on the recommendations of the Journal of the American Medical Association, we have used both terms with respectful and inclusive intent (Flanagin et al., 2021). Other terms such as First Peoples or Native peoples could include (but not limited to) Aboriginal and/or Torres Strait Islander, Maori, Native American or North American Indian or South American Indian, Inca, Inuk, Metis, and Inuit peoples. Papers were included that specifically included participants who identify as Indigenous and/or First Nations. Studies and textual reports that focused on culturally and linguistically diverse or ethnic minority populations that did not include Indigenous and/or First Nations people were excluded.

#### Setting

LTC facilities were defined as any “nursing and residential care facilities which provide accommodation and LTC as a package. This refers to specially designed institutions or hospital-like settings (e.g., nursing homes) where the predominant service component is LTC, and the services are provided for people with moderate to severe functional restrictions” (Royal Commission into Aged Care Quality and Safety, 2020, p. 10). Although the terms used vary from country to country, we included common terms such as residential care facilities, nursing homes, care homes, assisted living facilities, and homes for the aged. Studies and textual reports in settings providing acute or short-term care, such as hospital acute care wards or day respite care were excluded.

#### Phenomena of interest

Elements or models of care provided in LTC settings for Indigenous and First Nations peoples, including but not limited to, the physical and social environment, staff and resident mix, meeting everyday needs, involvement of the person and family in planning and implementing care, and philosophies underpinning care.

#### Outcomes of interest

QoL or consumer satisfaction for Indigenous and First Nations peoples, measured by validated tools, surveys, or qualitative data.

#### Types of studies and textual reports

Qualitative studies or studies of mixed methods design, including observational, cross-sectional, descriptive case studies, and action research studies were included. Systematic reviews were not included in this review as primary articles (as per the JBI guidelines), however, references were hand-searched to identify potential studies. We also included data from reports or policy documents, including guidelines, discussion or position papers, and other text relevant to answering the research question.

#### Search strategy

We used a three-step search strategy to find both published and unpublished studies. An initial search of specific Indigenous groups and aged or residential care and QoL or consumer satisfaction with related indexed terms and keywords in the title or abstract was conducted. However, as there were not enough results for a meaningful review, we used an amended search strategy of culturally appropriate care or services and Indigenous and First Nations peoples and aged or residential care. We did not use any specific Indigenous population group to ensure the results remained broad, however, some of the indexed terms included named groups. The lens or filter of QoL and/or consumer satisfaction were used as selection criteria. A second search using all identified keywords and index terms was then undertaken across included databases. Third, the reference list of included reports and articles was searched for additional studies. Studies published in the English language with no date restriction were considered for inclusion. The databases searched included: EMBASE, PsycInfo, CINAHL, Medline, Nursing and Allied Health, and Health and Medical Collection (Proquest). Australian Policy Online, National Aboriginal Community Controlled Health Organisation, and Google were also searched using the terms (“Aboriginal” OR “Indigenous” OR “Torres Strait Islander”) AND “aged care” to find government reports, policy documents, and other gray literature from Australia. For international gray literature, targeted searches of the United Nations, WHO, OECD iLibrary, Google, and government websites were undertaken (United States, Canada, New Zealand, and Finland). Names for the Indigenous peoples of these countries were used in conjunction with “aged care,” to make the results more manageable to scan and select.

Searches were run in October 2020, and rerun before final analyses in March 2021. Search results were saved to Endnote X9 and duplicates removed. The search strategies are reported in Supplementary Material S1.

### Selection and Critical Appraisal

The identified titles and abstracts were screened for eligibility by D. Brooks. Papers selected for full-text retrieval were screened for congruence with the inclusion criteria by two independent reviewers (D. Brooks and M. Brodie). Disagreements were resolved through adjudication by a third reviewer (M. MacAndrew). Studies excluded at this stage were recorded with reasons (Supplementary Material S2). Studies that met the inclusion criteria were assessed for methodological quality by four teams of two independent reviewers (C. Parker & D. Brooks, D. Brooks & M. Brodie, S. Johnston & D. Brooks, and M. MacAndrew & D. Brooks) using JBI standardized critical appraisal instruments and recorded in JBI SUMARI (Aromataris, 2020). Due to the paucity of research studies in this area, none were excluded on methodological quality but have been reported as high, moderate, or low confidence in the findings with respect to (a) dependability (methodological limitations) and (b) credibility (congruency between the author’s interpretation and the supporting data, rated as unequivocal, and credible or not supported) using the ConQual approach to qualitative evidence synthesized findings (Munn et al., 2014).

### Data Extraction and Synthesis

Qualitative data were extracted using the standardized data extraction tool from JBI SUMARI (Aromataris, 2020) and included details about (a) methods for data collection and analysis; (b) country; (c) the phenomena of interest; (d) setting/context/culture; (e) participant characteristics and sample size; and (f) description of main results of significance to the review question. For textual reports, the data extracted included: (a) type of text; (b) population represented; (c) the topic of interest; (d) setting/context/culture; (e) stated allegiance/position; and (f) description of main arguments/recommendations of significance to the review question.

Research findings were categorized based on similarity in meaning across all Indigenous and First Nations groups. The JBI approach to synthesizing the findings from qualitative studies and conclusions of textual or nonresearch studies requires reviewers to consider the credibility (logic and authenticity) of each paper; identify and extract the findings or recommendations from each paper; and to aggregate these as synthesized findings based on shared meanings. Initially, data from the textual reports and qualitative studies were synthesized separately. Each synthesis involved a three-stage process as recommended by the JBI guidelines (Aromataris, 2020) and shown in Table 1.

**Table 1.** Three-Stage Process of Data Extraction and Synthesis

Level of data extraction and synthesis	Description
Level 1: Extraction of findings	The extracted findings from each of the papers, relating to elements of care that promote quality of life for Indigenous and First Nations peoples living in LTC, were assembled into JBI SUMARI, accompanied by a specific illustration (direct quotation) from the original text. For each extracted finding, a level of credibility was allocated (unequivocal, credible, or not supported).
Level 2: Categorization of findings	A category is a brief description of a key concept arising from the aggregation of two or more similar findings and is accompanied by an explanatory statement that conveys the whole, inclusive meaning of a group of similar findings. Findings from the papers were categorized based on similarity in meaning.
Level 3: Synthesized findings	A synthesized finding is an overarching description of a group of categorized findings. Synthesized findings are expressed as statements that can be used to generate recommendations for policy or practice. As with categories, a description was created for each synthesized finding; an explanatory statement that conveys the whole, inclusive meaning of a group of similar categories.

*Notes*: LTC = long-term care; JBI = Joanna Briggs Institute.

Finally, we used a convergent integrated approach to assemble the textual data with the qualitative data, according to the JBI methodology (Lizarondo et al., 2020). Assembled data were pooled to produce a set of integrated findings in the form of action statements.

## Results

### Study Inclusion

**Figure 1.**
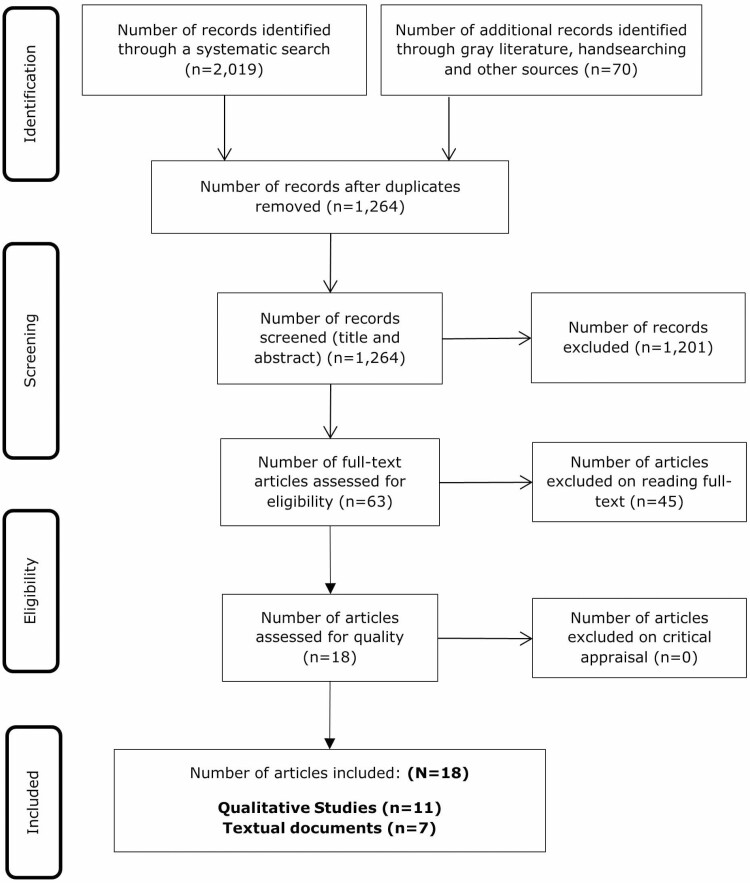
PRISMA flow diagram of search and selection process. PRISMA = Preferred Reporting Items for Systematic Reviews and Meta-Analyses.

A total of 2,019 papers were identified through the systematic database searches, and a further 70 papers through other sources. After duplicates were removed, 1,264 papers were screened by title and abstract. Of these, 63 papers were retrieved for full-text review by two reviewers (D. Brooks & M. Brodie); 45 papers did not meet the eligibility criteria and were excluded with reasons (Supplementary Material S2). Eighteen papers met the eligibility criteria and were assessed for methodological quality. The results of the study search and selection process are presented using the PRISMA flow diagram (Figure 1). The PRISMA checklist can be found in Supplementary Material S3.

### Study Characteristics

Whilst three mixed methods studies were included (Brown & Gibbons, 2008; Kataoka-Yahiro et al., 2016; Selle, 2007), the reported quantitative (survey) data was not relevant to this review question. Therefore, the data extracted relates to the qualitative components of mixed methods or purely qualitative studies (*n* = 11), and from textual reports/documents (*n* = 7). The study characteristics can be seen in Tables 2 and 3, respectively.

**Table 2.** Characteristics of Qualitative Studies

Study	Methods for data collection and analysis	Country	Phenomena of interest	Setting/context/culture	Participant characteristics and sample size	Description of main results
Brown and Gibbons (2008)	Mixed methods: survey and interviews. Thematic analysis.	USA	Compare the psychological well-being of residents of an ALF with nonresidents.	American Indian tribe; Rural area; Owned, managed, and funded by the tribe. Staff are both tribal and nontribal.	Survey of tribal members (n=56): ALF residents (*n* = 13); nonresidents (*n* = 43). Interviews with tribal members (*n* = 5).	Support provided by the staff; social support from the tribe and other residents; loneliness prevented by involvement in activities; relationship with other residents.
Du Toit et al. (2014)	Nominal group process. Content analysis.	South Africa	Explore how more meaningful and culturally relevant experiences could be facilitated for SeSotho Elders living in residential aged care.	Indigenous Elders in South Africa.	Staff members involved with the care of SeSotho Elders. (*n* = 15)	Helplessness (quality of care, autonomy and choice of residents, access, and functional abilities); Loneliness (belonging, cooccupations, and a sense of community); Companionship (and/or separation); and Boredom (doing, becoming, and environmental significance). Conclusion: “Human dignity and respect are upheld within residential care when Elders have access to purposeful and meaningful activities of their choice that are culturally appropriate.”
Hanssen and Kuven (2016)	Interviews. Interpretive analysis.	South Africa, Norway	To learn about the meaning of traditional food for residents with dementia.	Dementia care in South Africa, ethnic Norwegians, and the Sami in Norway.	Nurses (*n* = 13) and family members (*n* = 6).	Belonging and joy; improved appetite; food and reminiscence. Traditional foods created a feeling of belonging and joy. Familiar tastes and smells awoke pleasant memories and boosted sense of well-being, identity, and belonging.
Hendrix (2003)	Interview and focus group written up as a case study to illustrate the culturally congruent end of life care.	USA	The provision of health care and palliative care services for Native American Indians.	Native American Indian Elders in a hospice in the San Francisco area.	Native American Indian’s clinical nurse specialist, hospice physician, and brother.	Death was not spoken of directly. Staff asked for information about Navajo traditions and family preferences. Traditional healing and ceremonies as well as medical approach. Staff listened to the needs and preferences of the family for privacy and ritual and respect for the patient.
Kataoka-Yahiro et al. (2016)	Mixed methods. Content analysis for qualitative data.	Hawaii, USA	Asian and Native Hawaiian family satisfaction with palliative care services.	Asian and Native Hawaiian in nursing homes.	Family caregivers (*n* = 9).	Family caregiver satisfaction was related to a safe, supportive, and caring environment. Family caregiver and nursing home staff communication based on culturally appropriate approaches for palliative as well as end-of-life care services among Native Hawaiians.
Mercer (1996)	Interviews. Observation of care conferences, resident council meetings, and key staff meetings. Thematic analysis.	Arizona, USA	Exploration of culturally sensitive principles/practices that exist in the nursing home.	Navajo Nation nursing home (79 bed capacity) on a Navajo reservation.	Nursing staff (n = not stated); key administrative staff (n = not stated); Navajo Elders and family members (*n* = 10).	Communication; clan associations and social structure; personal space; modesty, cleanliness, and privacy; traditional foods; dying and death; cultural rituals. Cultural care practices influence all aspects of care services, including assessment, interventions, staff selection and training, policy and procedure implementation, facility location, and design.
Schultz and Helander (1988)	Interviews and environmental data. Descriptive analysis.	USA	Factors that affect the quality of life of Native American Alaskans when they move into long-term care.	Two hundred twenty bed not-for-profit long-term care facility, of which 20% of residents were Native Alaskan.	Staff members (*n* = 20).	Cultural indicators of adjustment to long-term care for Native Alaskans included: Diet; communication; personal care; interpersonal relationships; occupation and role; rituals and customs; attitudes toward health care.
Selle (2007)	Interviews. Thematic content analysis.	Alaska, USA	Explored access to traditional Native foods.	Alaskan Native Elders (Inuit) residing in Anchorage LTC facilities.	Care facility operators (*n* = 11) Alaskan Native Elders (*n* = 7); family members of Elders (*n* = 7).	Food as connection to culture. Native food is highly valued by residents. Lack of Native food affects mental health. Restrictions on Native foods unless family members provided it. Barriers include a lack of knowledge or understanding of Native foods by staff, communication difficulties between staff and residents, difficulty accessing Native foods or regulation issues.
Shomaker (1981)	Observations made in two Navajo nursing homes and interviews with personnel. Descriptive.	USA	Conflict of philosophies between traditional Navajo caring role and the western concept of institutionalization.	Navajo nursing homes, set within a reservation in Arizona.	Two nursing homes, staffed by Navajo workers and run by the Navajo Health Authority.	Activities to continue Navajo culture. Navajo food. Medicine men as well as physicians visit the residents. Residents are taken to Navajo events on reservation. A traditional “hogan” is available at each facility for ceremonial purposes
Sivertsen et al. (2019)	Interviews. Interpretive analysis, grounded in an Aboriginal- centered research approach.	Australia	Cultural care needs for Aboriginal residents from their own and carers’ perspectives.	Three residential aged care centers in South Australia, two rural and one urban metropolitan. Two facilities had 100% Aboriginal residents and one facility had 10%.	Aboriginal residents (*n* = 7) and formal carers (*n* = 19) within the facility.	Marginalization of Aboriginal culture within facilities. Residents showed a strong connection to identity, families, community, and land; staff were not aware of or did not place enough emphasis on the cultural needs of the Aboriginal residents to be included in the care provided; the presence of standards of care does not guarantee a culture-centered approach; the employment of more Aboriginal carers would result in a more culturally safe clinical environment.
Swinton (2011)	Interviews. Thematic analysis.	Eastern USA	Understanding the lived experiences of Native American Indian Elders. transitioning into LTC facilities.	Native American Indian Elders in LTC.	Family members (*n* = 20)	Family and community, familiar foods, and participating in ceremonies. Maintaining cultural ties for ensuring happiness and quality of life. Maintaining cultural practices, beliefs, community affiliation, ability to meet spiritual needs, and healing practices. Hire Indian staff members, support family involvement.

*Notes*: ALF = assisted living facility; LTC = long-term care.

**Table 3.** Characteristics of Textual Reports

Study	Type of text	Population represented	Topic of interest	Setting/ context/ culture	Stated allegiance/ position	Description of main argument(s)
Australian Government Department of Health (2019a)	Guide for consumers.	Aboriginal and Torres Strait Islander peoples.	A resource to support people working in aged care to understand the needs and perspectives of Aboriginal and Torres Strait Islander peoples.	Australian aged care.	The right to access quality, inclusive and culturally safe aged care that cater for the diverse characteristics and life experiences of older Australians.	Consumers should be active partners in planning and implementation of aged care systems; access to aged care services and support even if living in rural areas; should experience a flexible aged care system that is responsive to new and emerging communities, including an increasingly diverse aged care workforce; respectful and inclusive care; high quality, culturally safe care.
Australian Government Department of Health (2019b)	Guide for providers.	Aboriginal and Torres Strait Islander peoples.	Actions to support inclusive care for Aboriginal and Torres Strait Islander peoples.	Australian aged care.	Action plan addresses the challenges experienced by Aboriginal and Torres Strait Islander communities in accessing the aged care system and provides foundational actions.	Opportunities to continue participating in cultural events and activities; acknowledge cultural factors and life experiences could be responsible for challenging behaviors; understand the concept of Elders in Aboriginal culture; understand the importance of the role of informal caregivers; ensure managers complete cultural competency training to provide leadership and guidance to staff; develop policies and processes for identifying and reporting racism and discrimination; recognize and celebrate historical events of significance; ensure members of the Stolen Generation are supported by staff trained to provide trauma-informed care; ensure culturally safe spaces are made available to allow families and communities to come together; codesign facilities, services,
						and programs with local Aboriginal groups; work in partnership with organizations to develop culturally appropriate services for members of the Stolen Generations.
Health Quality & Safety Commission New Zealand (2019)	Aged care profile.	Maori	Māori principles of care in aged care plans.	Kowhainui Village in Whanganui, New Zealand (residential aged care).	Provider organization as an example of good practice within New Zealand.	Description of a new model of care for Māori aged care residents with “Te Whare Tapa Whā” as a key principle. The cultural and spiritual needs of new residents and their whānau (families) must be met with respect and consideration. An advisory group includes whānau (relatives, community), residents, staff, cultural advisors, and kaumātua (a maori Elder). The work aims to combine both the four elements of Te Whare Tapa Whā with elder-directed model of care (the Eden Alternative).
National Aboriginal Community Controlled Health Organisation [NACCHO] (2019)	Report (Submission to Royal Commission into Aged Care Quality and Safety)	Aboriginal and Torres Strait Islander peoples.	How best to deliver aged care services in a sustainable way, including through innovative models of care and investment in the aged care workforce	Australian aged care for Aboriginal and Torres Strait Islander older adults.	The report includes feedback received from member services, ACCHOs, who deliver a range of services in urban, rural, and remote communities across Australia.	Cultural safety be embedded across all areas of aged care and a mandatory part of accreditation processes. Mainstream aged care services commit to work collaboratively with local ACCHOs, including seeking their advice on issues relating to cultural safety and trauma- informed care. ACCHOs be funded to deliver regular cultural competency training to mainstream aged care providers. Funding for interpreters be available for Aboriginal and Torres Strait Islander language speakers.
						ACCHOs are designated as preferred providers of primary health care for all Aboriginal and Torres Strait Islander residents of aged care facilities. Aged care services are funded to employ Aboriginal liaison officers.
National Advisory Group for Aboriginal and Torres Strait Islander Aged Care [NAGATSIAC] (2019)	Report (Submission to the Royal Commission into Aged Care Quality and Safety)	Aboriginal and Torres Strait Islander peoples.	Recommendations for change to ameliorate the problems older Aboriginal and Torres Strait Islander people and communities face when trying to access aged care support and receive culturally safe aged care services.	Australian aged care services for Aboriginal and Torres Strait Islander older adults.	The Aboriginal and Torres Strait Islander model of holistic, community-centered care that is trauma- informed, and provides wrap-around services to community members on-site.	Mainstream providers should partner with Aboriginal and Torres Strait Islander health services. Accreditation and funding should be dependent on the provision of cultural safety training and trauma-informed care training for management and staff, facilitated by Aboriginal and Torres Strait Islander families and community-controlled service providers. Aboriginal access to Person Centered Care requires its delivery in an Aboriginal involvement framework of family/ community/kin. Mainstream providers to employ more Aboriginal and Torres Strait Islander staff. Stolen Generations survivors to age on Country, with mob/other Aboriginal and Torres Strait Islander community members who may be from different communities. Codesign service delivery for survivors of the Stolen Generations with Stolen Generations descendants, and Aboriginal and Torres Strait Islander community members.
Office of Evaluation and Audit [OEA] (2009)	Report	Aboriginal and Torres Strait islander peoples.	Performance audit of residential aged care services for Indigenous peoples.	Australian residential aged care.	Commonwealth of Australia report to address Aboriginal and Torres Strait Islander cultural quality of care in residential care settings.	Culturally appropriate care includes: arts and crafts activities for residents; providing traditional foods; encouraging visits by family members to the facility; attending cultural ceremonies; increasing knowledge on “sorry business”; Understanding kinship systems and the way it may affect the provision of care; encouraging community input on governing the facility, activities, and celebrations; training and employment of local Indigenous workers is important in delivering effective aged care services in remote areas.
Regional Development Council of Western Australia [RDCWA] (2016)	Report	Older residents in regional Western Australia.	Identification of key issues affecting aged care in regional Western Australia and potential solutions; focus on the delivery of appropriate care for older Aboriginal peoples.	Regional Western Australia.	Differing cultural expectations between Aboriginal and non-Aboriginal communities mean nonstandard design features are required for building facilities.	Preference for community care as opposed to entering residential aged care; however, problems exist with appropriate housing in which to deliver care; remote communities are a challenge to delivering care; high prevalence of dementia and cognitive impairment; residential aged care needs to be capable of accommodating greater family presence; culturally safe care.

*Note*: ACCHO = Aboriginal Community Controlled Health Organization.

#### Setting/context/culture

For the qualitative studies, eight were conducted in the United States (Brown & Gibbons, 2008; Hendrix, 2003; Kataoka-Yahiro et al., 2016; Mercer, 1996; Schultz & Helander, 1988; Selle, 2007; Shomaker, 1981; Swinton, 2011), one in Australia (Sivertsen et al., 2019), one in South Africa (Du Toit et al., 2014), and one included data from both South Africa and Norway (Hanssen & Kuven, 2016). The Indigenous and First Nations perspective explored included American Indian Elders (Navajo; Brown & Gibbons, 2008; Hendrix, 2003; Mercer, 1996; Shomaker, 1981; Swinton, 2011), Alaskan Elders (Inuit; Schultz & Helander, 1988; Selle, 2007); Hawaiian Elders (Kataoka-Yahiro et al., 2016), South African Elders (So Sotha; Du Toit et al., 2014), Norwegian Elders (Sami; Hanssen & Kuven, 2016), and Australian Aboriginal Elders (Sivertsen et al., 2019). Only one of the studies’ authors identified as being an Indigenous and First Nations researcher (Sivertsen et al., 2019), with the others not stated.

For textual reports, six were related to Aboriginal and Torres Strait Islander peoples within the Australian aged care system (Australian Government DoH, 2019a, 2019b; National Aboriginal Community Controlled Health Organisation [NACCHO], 2019; National Advisory Group for Aboriginal and Torres Strait Islander Aged Care [NAGATSIAC], 2019; Office of Evaluation and Audit [OEA], 2009; Regional Development Council of Western Australia [RDCWA], 2016) and one was related to Māori peoples residing in LTC in New Zealand (Health Quality & Safety Commission New Zealand [HQSCNZ], 2019). Of these, three indicated that they were written by or with input from First Nations peoples (Australian Government DoH, 2019a; NACCHO, 2019; NAGATSIAC, 2019).

#### Phenomena or topic of interest

The phenomena of interest addressed in the qualitative studies all encompassed elements of care that promote well-being, QoL, and satisfaction for Indigenous and First Nations people living in LTC, however, some had a particular focus. For example, the provision of culturally appropriate palliative care for Indigenous Elders (Hendrix, 2003; Kataoka-Yahiro et al., 2016); access to and the meaning of Indigenous traditional food within LTC (Hanssen & Kuven, 2016; Selle, 2007); meaningful and culturally relevant experiences for First Nations people in LTC (Du Toit et al., 2014; Swinton, 2011); cultural care needs (Sivertsen et al., 2019); culturally sensitive principles, philosophies, and care practices (Mercer, 1996; Shomaker, 1981); and factors influencing QoL (Brown & Gibbons, 2008; Schultz & Helander, 1988).

The topic of interest addressed in most of the textual reports was the provision of inclusive, culturally safe, and appropriate aged care services for Australian Aboriginal and Torres Strait Islander peoples (Australian Government DoH, 2019a, 2019b; NACCHO, 2019; NAGATSIA, 2019; OEA, 2009; RDCWA, 2016). One document focused on the inclusion Māori principles of care in LTC (HQSCNZ, 2019).

#### Methods for data collection and analysis

Semistructured interviews (*n* = 10; Brown & Gibbons, 2008; Hanssen & Kuven, 2016; Hendrix, 2003; Kataoka-Yahiro et al., 2016; Mercer, 1996; Schultz & Helander, 1988; Selle, 2007; Shomaker, 1981; Sivertsen et al., 2019; Swinton, 2011); a focus group (Hendrix, 2003); and observations within the LTC facility (Mercer, 1996; Shomaker, 1981) were used to collect data. One study used a nominal group process where opinions were discussed and priorities ranked by an expert group (Du Toit et al., 2014). Data were analyzed using mainly thematic content analysis (Brown & Gibbons, 2008; Du Toit et al., 2014; Kataoka-Yahiro et al., 2016; Mercer, 1996; Schultz & Helander, 1988; Selle, 2007; Swinton, 2011) or interpretive descriptive analysis (Hanssen & Kuven, 2016; Sivertsen et al., 2019). However, one study was descriptive rather than analytical (Shomaker, 1981), and another reported data as a case study (Hendrix, 2003).

#### Participant characteristics and sample size

The sample size for the qualitative studies ranged from five interviewees (Brown & Gibbons, 2008) to 26 (Sivertsen et al., 2019); two studies did not state how many interviews were conducted (Hendrix, 2003; Shomaker, 1981). At least 159 participants were interviewed across the nine studies. Of these, 78 were staff members, 52 were family members, and 29 were Indigenous Elders (residents). Age and gender of participants were not routinely reported, however, of those that did, most (62%) participants were female, and age ranged from 49 to 77 years for family carers and 58–96 years for residents.

### Quality Assessment

For most of the qualitative studies, there was congruity between the research methodology and the research question and data collection methods, and between the research question and the presentation and analysis of the data. Less than half (45%) of the studies stated their philosophical perspective, and only one study located the researcher culturally or theoretically. Ethical approval was not always stated in the papers. Few assessed the influence of the researcher on the research process and interpretations. Due to the varying nature of reporting, the representation of participants’ voices was mixed, however, for most studies, the conclusions appeared to flow from the analysis or interpretation of data. The credibility of findings was unequivocal for most of the studies, and there was congruency between the author’s interpretation and the supporting data (Munn et al., 2014). The quality of the textual reports was high in terms of the source of opinion and standing of that source in the field, as well as representing the interests of Indigenous and First Nations populations. However, not all were clear about the basis of their recommendations/arguments or whether these arguments were analytical. Most documents did not provide supporting references from the literature. Methodological quality can be seen in Supplementary Material S4.

### Synthesized Findings

The following four synthesized findings were identified that described elements or models of care that may help to promote QoL for Indigenous and First Nations people in LTC. The categories and synthesized findings, with illustrative examples, are summarized in Table 4.

**Table 4.** Categories and Synthesized Findings

Synthesized finding (level 3)	Category (level 2)	Illustrated examples of findings (level 1)	Studies reporting finding	Level of credibility
1. LTC services should be codesigned and provided in collaboration with Indigenous and First Nations communities and organizations to help ensure culturally safe care and to recruit and retain local Indigenous workers	Codesign and collaboration with Indigenous and First Nations communities and organizations	“Co-design facilities, services and programs with local Aboriginal groups and work in partnership with organisations that have necessary expertise to develop culturally appropriate and responsive services for vulnerable consumers such as members of the Stolen Generation.” DoHa p. 13 “We all want to access culturally safe aged care services, whether we live in a rural, regional or metropolitan area. We are able to live on Country. Our providers involve members of our communities in the design and delivery of some of the service in our care plan.” DoHb p. 5	DoHa DoHb RDCWA NAGATSIAC NACCHO OEA Shomaker	Unequivocal
	Culturally appropriate design of LTC homes and grounds	“The facility was built expressly for extended care use and though a new and modern building is in the hexagonal shape of a traditional Hogan.” Shomaker p. 532 “Differing cultural expectations between Aboriginal and non- aboriginal communities mean that nonstandard design features are called for in building facilities for Aboriginal older people. The first is in respect of design for residents where the preference is likely to be for open community style accommodation.” RDCWA p. 9	DoHa Mercer RDCWA Shomaker	Unequivocal
	Indigenous care providers and staff	“Culturally safe and trauma informed healing focused aged care service provision can best be provided by Aboriginal and Torres Strait Islander aged care service providers.” NAGATSIAC p. 22 “The Navajo nursing homes are owned and operated by the tribe allowing Navajos to care for their elderly within their cultural resources.” Shomaker p. 534	NACCHO NAGATSIAC OEA Shomaker Sivertsen Swinton	Unequivocal
2. LTC facilities need to embed trauma- informed care policies, practices, and staff training to deliver culturally safe services to members of the Stolen Generation	Cultural training	“Ensure managers employed by providers with 5% or more Aboriginal and Torres Strait Islander consumers complete an advanced level of cultural competency training to position them to provide leadership and informed guidance to staff.” DoHa p. 12 “Aboriginal community-controlled organisations be funded to deliver regular cultural competency training, tailored to local protocol, to mainstream aged care providers.” NACCHO p. 15	DoHa Du Toit NACCHO Sivertsen Swinton	Unequivocal
	Trauma-informed care training, practices, and policies	“As 100% of the Stolen generation will be aged 50 by 2023 we require an aged care system that is aware of the harmful impact of colonization and the trauma caused by removing children from families.” DoHb p. 5 “Training for aged care staff, Indigenous and non-Indigenous, on healing and trauma informed approaches to ensure they are embedded in all aspects of service provision.” NAGATSIAC p. 13	DoHa DoHb RDCWA NAGATSIAC NACCHO	Unequivocal
3. LTC facilities should meet the individual needs of Indigenous and First Nations residents in a culturally respectful way that includes upholding cultural, spiritual and religious beliefs, activities, and practices, including those surrounding end of life	Culturally appropriate and meaningful activities and events	“Recognise and celebrate historical events of significance and important annual events e.g. Close the Gap, Mabo Day, as a normal part of business.” DoHa p. 12 “Activities are designed to continue familiar patterns of Navajo culture e.g., weaving, jewelry making, pottery, squaw dances.” Shomaker p. 532	DoHa DoHb Du Toit Schultz Shomaker Sivertsen	Credible Unequivocal
	Cultural needs, spiritual and religious beliefs, and practices	“Personal space is important to Navajos, and some have difficulty adapting to spaces and people with whom they are not familiar. Traditional Navajos sleep on mattresses or sheep skins stacked on the floor. The Grandparents were not accustomed to the high hospital bed with railings and often wanted to move the mattress to the floor, a request honored by staff.” Mercer p. 187 “The importance of aged care providers recognising, respecting and making provisions for upholding older Aboriginal and Torres Strait Islander people’s cultural values, beliefs and practices regarding traditional foods and food taboos, gender, clan and kinship laws, and ceremonies and taboos relating to death and dying.” NACCHO p. 6	DoHb Du Toit Hanssen Hendrix HQSC Mercer NACCHO OEA Schultz Selle Shomaker Swinton	Credible Unequivocal
4. Culturally safe LTC includes the sustained involvement of family, kin, and the wider Indigenous community to maintain connection to culture and sense of belonging	Connection with other Indigenous and First Nations residents, families, kin, and community enhances the sense of belonging and reduces loneliness	“SeSotho elders who are bedbound or who have to share a room with someone from a different culture would experience separation and even segregation, which is in contrast to the Ubuntu worldview.” Du Toit p. 132 “They can visit and establish relationships with other older persons from the reservation and they are living on the reservation being cared for by Navajos who speak Navajo.” Shomaker p. 532	Brown Du Toit Schultz Shomaker Swinton	Credible Unequivocal
	Involvement of residents and families in culturally appropriate person-centered care and planning	“There was a desire from staff, residents and whānau to take Māori principles of care into consideration.” HQSC p. 1 “Aboriginal access to person-centred care requires its delivery in an Aboriginal framework of family/community/kin involvement.” NAGATSIAC p. 8	DoHb HQSC Kataoka-Yahiro NAGATSIAC OEA	Unequivocal
	Involvement of families and communities in maintaining connection to culture	“Community centred care is needed to protect the vital role of Elders as custodians of culture.” NAGATSIAC p. 22 “The elders need to stay close to their culture … Keeping ties with the family, clan members and receiving news from the community has to continue.” Swinton p. 102	Mercer NAGATSIAC Schultz Swinton	Credible Unequivocal

Finding 1. LTC should be codesigned and provided in collaboration with Indigenous and First Nations communities and organizations to help ensure culturally safe care and to recruit and retain local Indigenous workers.

#### Codesign and collaboration with Indigenous and First Nations communities and organizations

Six textual reports from Australia (Australian Government DoH, 2019a, 2019b; NACCHO, 2019; NAGATSIAC, 2019; OEA, 2009; RDCWA, 2016) and one qualitative study from North America (Shomaker, 1981) recommended the codesign of LTC facilities and collaboration with Indigenous communities and organizations to ensure culturally appropriate and safe services. This approach was considered best practice, particularly when designing trauma-informed care and services for members of the Stolen Generation in Australia (Australian Government DoH, 2019a, 2019b; NAGATSIAC, 2019). Developing close ties with the local Indigenous community was felt to be important in providing quality care (OEA, 2009). The study by Shomaker (1981), described the importance of having LTC facilities on the Navajo (North American Indian) reservation, run by the Navajo Health Authority and being cared for by people from their own culture. The importance of having the choice to live and die “on Country” was also discussed with reference to Indigenous Australians (Australian Government DoH, 2019a; NACCHO, 2019; NAGATSIAC, 2019; RDCWA, 2016).

#### Culturally appropriate design of LTC homes

The importance of a culturally appropriate LTC home design was discussed in two textual reports from Australia (Australian Government DoH, 2019b; RDCWA, 2016) and two research studies from North America (Mercer, 1996; Shomaker, 1981). In Australia, this approach included the interior design of the facility, for example, spaces for large family groups to meet and stay (RDCWA, 2016), open community-style accommodation (RDCWA, 2016), and culturally specific artwork (Australian Government DoH, 2019b). In Navajo facilities, this included the physical design of the building in the style of a traditional “hogan” (dwelling; Mercer, 1996), or the building of a traditional hogan next to the facility for use by residents and their families (Shomaker, 1981).

#### Indigenous care providers and staff

Three textual reports from Australia (NACCHO, 2019; NAGATSIAC, 2019; OEA, 2009), and three studies from North America (Shomaker, 1981; Swinton, 2011) and Australia (Sivertsen et al., 2019) concluded that Indigenous and First Nations care providers and staff were best placed to provide culturally safe and trauma-informed care to Indigenous and First Nations residents. In Australia, Aboriginal community-controlled organizations were identified as being ideally placed to be the direct providers of LTC for Aboriginal and/or Torres Strait Islander people, and it was recommended that long-term funding be increased and provided to do so (NAGATSIAC, 2019). Where Indigenous and First Nations residents are cared for in mainstream (non-Indigenous) facilities, it was recommended that an Indigenous liaison officer or “cultural advisor” be employed to work with residents and staff (NACCHO, 2019). The lack of Indigenous staff in mainstream facilities was thought to lead to marginalization, with little emphasis on Indigenous culture and activities (Sivertsen et al., 2019).

Finding 2. LTC facilities need to embed trauma-informed care policies, practices, and staff training to deliver culturally safe services to members of the Stolen Generation.

#### Cultural training

The need for cultural training (which was not defined in the papers), to be delivered to non-Indigenous care provider managers and staff was recommended in two textual reports from Australia (Australian Government DoH, 2019b; NACCHO, 2019) and three studies from South Africa (Du Toit et al., 2014), Australia (Sivertsen et al., 2019), and North America (Swinton, 2011). The NACCHO (2019) recommended that this training be provided by Indigenous organizations to mainstream providers. In Sivertsen et al. (2019), residents living in mainstream facilities and their families felt that staff lacked adequate cultural training and that there were few staff with knowledge of Indigenous health and cultures. This lack of knowledge often related to language difficulties but also knowledge of “Aboriginal Australia.”

#### Trauma-informed care training, practices, and policies

Related to the importance of cultural training was the need for specific trauma-informed care training in Australia (Australian Government DoH, 2019a, 2019b; NACCHO, 2019; NAGATSIAC, 2019; RDCWA, 2016). By 2023, 100% of Australia’s Stolen Generation will be aged 50 years of age or above and will need to be cared for by trained staff “who understand the risks of re-traumatizing us survivors and the meaning of healing” (Australian Government DoH, 2019a). It was recommended this training should be provided to all Indigenous and non-Indigenous LTC staff, to ensure adequate practices and policies are embedded across all aspects of service provision (NAGATSIAC, 2019).

Finding 3. LTC facilities should meet the individual needs of Indigenous and First Nations residents in a culturally respectful way that includes upholding cultural, spiritual and religious beliefs, activities and practices, including those surrounding end of life.

#### Culturally appropriate and meaningful activities and events

The recognition and celebration of significant Indigenous events and participation in Indigenous activities were recommended by two textual reports from Australia (Australian Government DoH, 2019a, 2019b) and four studies from South Africa (Du Toit et al., 2014), Alaska (Schultz & Helander, 1988), North America (Shomaker, 1981), and Australia (Sivertsen et al., 2019). In Australia, significant events included Mabo Day on June 3rd, a commemorative day that occurs during National Reconciliation Week and which acknowledges the high court decision to recognize the precolonial land ownership of Indigenous Australians within Australia’s common law in the hybrid form of title called, Native Title. Culturally appropriate activities such as Indigenous music, dance, storytelling, games, crafts, weaving, and jewelry making were recognized as being important for Indigenous and First Nations peoples in papers from South Africa (Du Toit et al., 2014), Alaska (Schultz & Helander, 1988), North America (Shomaker, 1981), and Australia (Sivertsen et al., 2019).

#### Cultural needs, spiritual and religious beliefs, and practices

Three textual reports from Australia (Australian Government DoH, 2019a; NACCHO, 2019; OEA, 2009) and six studies conducted in South Africa (Du Toit et al., 2014), North America (Hendrix, 2003; Mercer, 1996; Shomaker, 1981; Swinton, 2011), and Alaska (Schultz & Helander, 1988) identified the importance of meeting the cultural and spiritual needs of Indigenous and First Nations residents. Cultural needs included culturally appropriate clothing (Du Toit et al., 2014), personal space, privacy and sleeping preferences (Mercer, 1996), communication in the language of choice (Du Toit et al., 2014; Mercer, 1996), bathing and toileting norms (Schultz & Helander, 1988), and provision of care by a member of the same gender (Schultz & Helander, 1988).

Six studies from South Africa/Norway (Hanssen & Kuven, 2016), North America (Mercer, 1996; Shomaker, 1981; Swinton, 2011), and Alaska (Schultz & Helander, 1988; Selle, 2007) discussed the importance of traditional food for the mental well-being and physical health of Indigenous and First Nations residents. Traditional food, tastes, and smells were said to invoke memories and encourage interaction and storytelling among residents, some of whom had dementia and did not usually speak (Hanssen & Kuven, 2016; Swinton, 2011). Lack of access to traditional foods was thought to negatively impact residents, both mentally and physically (Selle, 2007). Selle (2007) found that LTC facilities in Alaska did not typically serve traditional foods and that access was limited to family and friends bringing food into the facility.

The inclusion of and respect for traditional healing practices and access to traditional healers were also identified as important in three studies from North America (Hendrix, 2003; Shomaker, 1981; Swinton, 2011). There was a specific reference to spiritual and religious practices and the importance of respecting and upholding end-of-life traditions and ceremonies in North America (Hendrix, 2003; Mercer, 1996) and Australia (NACCHO, 2019; OEA, 2009).

Finding 4. Culturally safe LTC includes the sustained involvement of family, kin, and the wider Indigenous community to maintain a connection to culture and a sense of belonging.

#### Connection with other Indigenous and First Nations residents, families, kin, and community enhances sense of belonging and reduces loneliness

Three studies from North America (Brown & Gibbons, 2008; Swinton, 2011) and South Africa (Du Toit et al., 2014) discussed how connection to other Indigenous and First Nations residents, kin, and community members enhanced a feeling of belonging and reduced feelings of loneliness. Du Toit et al. (2014) described how Elders from SeSotho (South Africa) experienced feelings of separation or segregation if they did not share a room with someone from their own culture and in keeping with their “Ubuntu” worldview (a belief in a universal bond of sharing that connects humanity). It was recommended that younger residents from the same culture helped care for frail Elders and that children be enabled to visit the facility to listen to stories told by Elders (Du Toit et al., 2014). The importance of connection to family was also recommended to reduce feelings of loneliness (Swinton, 2011).

#### Involvement of residents and families in culturally appropriate person-centered care planning

It was recommended in four textual reports from Australia and New Zealand (Australian Government DoH, 2019a; HQSCNZ, 2019; NAGATSIAC, 2019; OEA, 2009) and one study from Hawaii (Kataoka-Yahiro et al., 2016), that residents and their families be involved in culturally appropriate person-centered care planning. In Australia, person-centered care delivery was described as being within a framework of family/community/kin involvement (NAGATSIAC, 2019). Residents were said to want a chosen family member present when making decisions about their care (Australian Government DoH, 2019a). In New Zealand, there was a desire from staff, residents, and whānau (extended family) to take Māori principles of care into consideration, including the four elements of Te Whare Tapa Whā (te tahu wairua [spiritual well-being]; te taha hinengaro [mental and emotional well-being]; te taha tinana [physical well-being]; and te taha whānau [family and social well-being]).

#### Involvement of families and communities in maintaining connection to culture

Three studies from North America (Mercer, 1996; Swinton, 2011) and Alaska (Schultz & Helander, 1988) discussed the importance of families and communities in maintaining connection to culture. Kinship and clanship are also regarded as important in many cultures (Mercer, 1996; Schultz & Helander, 1988). Swinton (2011) reported how keeping ties with family, clan members and receiving news from the community was important for Native American residents. One Australian report discussed the importance of respecting and supporting the role and responsibilities of Elders as custodians of culture (NAGATSIAC, 2019).

### Summary of Review Findings (Conqual)

Based on the methodological quality and credibility of the findings, we have moderate confidence in the synthesized findings of this review (Supplementary Material S5).

## Discussion

This review is the first we could find to identify elements or models of care that could promote QoL for Indigenous and First Nations peoples residing in LTC. This review included papers that focused on Indigenous and First Nations peoples worldwide and while the qualitative studies were conducted mostly in the United States and the reports were mainly from Australia, the congruence of elements promoting QoL was evident across all Indigenous and First Nations groups.

The specific and interrelated elements or models of care that promote QoL or consumer satisfaction for Indigenous and First Nations peoples living in LTC identified in this review reflect and extend the QoL factors identified by the “Good Spirit, Good Life” framework, which were not specific to LTC or other Indigenous and First Nations peoples globally (Smith et al., 2020). We will discuss each of these findings in turn.

### Codesign and Collaboration With Indigenous and First Nations Communities and Organizations to Promote Culturally Safe Care

The experience of Indigenous and First Nations peoples residing in LTC (and perceptions of LTC service providers and family members) is affected by services designed to cater to Western culture, and ill-equipped to cater to their cultural needs. This finding is consistent with those from a review exploring Indigenous Māori people’s experiences within LTC, where nonalignment with Māori cultural values and lack of cultural diversity in the health care workforce impacted on the care provided and uptake of services (Keelan et al., 2021).

The concept of health in Indigenous groups is broader than just physical health. In the Māori model of health, for example, health is likened to the four walls of a house and includes the physical (tinana), mental and emotional (hinengaro), family (whanau), and spiritual (wairua) realms (Rolleston et al., 2020). Similarly, Aboriginal and Torres Strait Islander peoples describe health as including physical, social, emotional, and cultural well-being of the whole community (National Aboriginal Health Strategy Working Party, 1989 as cited in Best & Fredericks, 2018, p. 15) and American Indians place emphasis on the essence of “spirit” in determining health (Smyer & Stenvig, 2007). Compared with the WHO view of health determinants (WHO, 2017), the Indigenous perspective places equal emphasis on the spiritual and cultural aspects as well as the inclusion of a community perspective, rather than focusing on biomedical models.

Models of aged care for Indigenous and First Nations peoples need to be established in a genuine partnership with local communities, incorporate culture specific to a particular community into the operation of the service and involve community members in a meaningful and ongoing capacity (Lindeman et al., 2017). Genuine partnerships between Indigenous communities and service planners that allows a process of identification of suitable and flexible approaches to care provision results in locally appropriate, sustainable services in both urban (Dance et al., 2004) and remote (Smith et al., 2010) settings. The process of developing the model of care is a key element in service development and delivery (Dance et al., 2004; Smith et al., 2010). However, published findings regarding Indigenous models of care are limited by the Western lens placed on the review of academic publications (Rolleston et al., 2020). As a result, service development tailored for Indigenous and First Nations peoples is compromised (Rolleston et al., 2020).

### Embedding Trauma-Informed Care Policies and Practices, and Staff Training to Deliver Culturally Safe Services

A suitably skilled workforce equipped with skills, attitudes, and knowledge to ensure culturally appropriate care was seen as critical to achieving high-quality and culturally safe care for Indigenous or First Nations people in LTC. Workers involved in service delivery must receive adequate education and training in Indigenous history, culture, specific local knowledge, and necessary communication skills, tied to the needs and perspectives of the local population (Clapham, 2017). While some of the reviewed papers talked about “cultural competency training,” no definition of what was meant by this “cultural competency” in Indigenous matters was provided. The most commonly used definition of cultural competence is “a set of congruent behaviors, attitudes, and policies that come together in a system, agency, or among professionals and enable that system, agency, or those professionals to work effectively in cross-cultural situations” (Cross, 1989, p. 7). In comparison, cultural safety addresses the notion of power and the impact of power differentials on care and is described as providing “a focus for the delivery of quality care through changes in thinking about power relationships and patients’ rights” (Papps & Ramsden, 1996, p. 493). While it can be argued that it is both impossible and culturally unsafe to teach “competency” in other peoples’ cultures, cultural safety training education that focuses on the history, current social circumstances and matters of power and dominance such as racism, should be provided (Cox et al., 2021). Education needs to be actioned by having structural resourcing and support for ongoing cultural safety critically reflective practice on clinical encounters and their outcomes by staff and services (Cox & Best, 2022). This is supported by the Australia Government (2019, p. 5), which states, “Cultural safety must become embedded across health services, particularly within the mainstream sector.” It is crucial, therefore, that those providing LTC services for Indigenous and First Nations peoples, are knowledgeable about historical and contemporary institutionalized racism, traumatic relationships, and treatments at the hands of health and all government services.

In Australia, cultural safety is embedded in professional nursing’s regulatory framework (Australian Nursing and Midwifery Accreditation Council, 2019; Nursing and Midwifery Board of Australia, 2018) and is also reflected in New Zealand and Canadian Nursing Council’s standards and competencies (Canadian Nurses Association, 2022; Nursing Council of New Zealand, 2011). However, cultural safety is not embedded in American nursing professional standards, which remains a barrier for improving health equity, especially for American Indian and Alaskan Native peoples (Pool & Stauber, 2020). In keeping with the principles and practice of the cultural safety model (Cox et al., 2021), health professionals and systems need to acknowledge their part in genocide, colonization, and neo-colonization in concert with other agents of the state, such as police, welfare, and education systems. Forsyth (2007) describes the role of nurses in implementing policy and practices that cemented and enacted institutionalized racism, and the extension of segregation and interiorization of Indigenous Australians in hospitals. If LTC staff are unaware of these matters, building rapport and trusting relationships, the basis of providing culturally safe holistic and quality care, will remain elusive.

Equally important in developing a culturally safe non-Indigenous workforce is recruitment, retention, and training of an Indigenous aged care workforce, thereby addressing cultural barriers, including trust, which Indigenous and First Nations Elders may experience (Cox, 2007). Indigenous and First Nations workers are considered part of the community and, therefore may have a different kin-based relationship with residents (Clapham, 2017). Currently, in Australian LTC facilities, less than 2% of direct care workers identify as Indigenous or First Nations (Jongen et al., 2019). Barriers to Indigenous and First Nations health workforce recruitment and retention include limited organizational funding, limited career pathways, and lack of mentoring (Gwynne & Lincoln, 2017). The development and sustainability of an Indigenous and First Nations workforce is therefore an important strategy, with emphasis on the importance of Indigenous and First Nations workers in leadership roles within organizations (Taylor et al., 2020).

### Being Respectful of Individual Needs, and Upholding Cultural, Spiritual and Religious Beliefs, Traditional Activities, and Practices

Person-centered care approaches are frequently espoused as best practice within the LTC literature (Thompson et al., 2018) and are associated with contributing to good QoL (Ballard et al., 2018), however, this concept has not been explored fully within the context of caring for Indigenous and First Nations peoples. Deravin et al. (2021) identify that while the Australian Aged Care Quality and Safety Commission refers to the varied social, cultural, linguistic, religious, spiritual, psychological, medical and care needs, characteristics and life experiences of aged care consumers, little to no reference is made about the specific needs of Indigenous Australian peoples. Identification and consideration of the specific needs, beliefs, and practices that are important to individuals who identify as belonging to a particular Indigenous or First Nations people should be recognized as best practice for culturally safe person-centered LTC (Deravin et al., 2021; Simonds et al., 2013). This includes consideration of appropriate and meaningful activities and events, clothing, personal space, privacy and sleeping preferences, language of choice, traditional foods, healing, and end of life practices.

### Promoting Connection to Culture and Sense of Belonging Through Sustained Connection With Family, Kin and Indigenous and First Nations Communities

Connection to family, kin, community, and Country have previously been identified as important aspects of QoL and as a means of connecting to culture (Brown & Gibbons, 2008; Butler et al., 2019; Sivertsen et al., 2019; Smith et al., 2020; Swinton, 2011). This connection is deemed vital for the enhancement of “feelings of belonging” and reduction of “feelings of loneliness,” especially for those residing within non-Indigenous care facilities. The reframing of person-centered care delivery within the framework of family/community/kin involvement is also of key importance to Indigenous and First Nations peoples within LTC.

### Limitations

This study was limited to papers in the English language and access to databases. The quality assessment process helped understand the strengths and weaknesses of the evidence, however, it was not possible to report rigorous outcomes due to the methodological variations in the studies. Based on the ConQual approach to synthesized findings, we have moderate confidence in the evidence presented. Most of the studies reported the perspectives of staff or family members, and further studies are needed that include the perspectives of Indigenous and First Nation LTC residents on models of care that promote their QoL. Furthermore, only one qualitative study (Sivertsen et al., 2019) and three reports (Australian Government DoH, 2019a; NACCHO, 2019; NAGATSIAC, 2019) identified their authorship as being by or with Indigenous and First Nations people. Therefore, it could be reasonably assumed that most studies are from a non-Indigenous perspective raising questions about the degree to which Indigenous peoples themselves were partners in the work, and consequently placing the utility of their recommendations in doubt.

## Conclusion

This review has extended our understanding of elements of care that contribute to good QoL for Indigenous and First Nations people living in LTC, however, there is a paucity of literature using quantitative measures of QoL. While the review has highlighted the uniqueness of Indigenous populations worldwide, there are common elements that are regarded as necessary for good QoL for Indigenous and First Nations people living in LTC: codesign and collaboration to promote culturally safe care; policy and staff training to enact trauma-informed care and culturally safe services; respecting cultural individuality, traditional activities, and practices; and connection to culture, family and kin. These findings could inform a model of care development specific to Indigenous and First Nations people in LTC.

## Supplementary Material

gnac153_suppl_Supplementary_Materials

## Transparency and Openness

This systematic review was conducted according to a priori protocol registered in PROSPERO (CRD42020218542). The review followed the Preferred Reporting Items for Systematic Reviews and Meta-Analyses (PRISMA) reporting guidelines (Page et al., 2021), the Joanna Briggs Institute (https://jbi.global/) methods for systematic reviews (Aromataris, 2020; Lizarondo et al., 2020) and the ConQual approach to assessing confidence in qualitative evidence synthesized findings (Munn et al., 2014). Data were extracted using the standardized data extraction tool from JBI SUMARI (Aromataris, 2020). The search terms and strategies are provided in the Supplementary Material to allow for replication. The authors have included the PRISMA checklist.
