# Supplementary material for: Elements of Long-Term Care That Promote Quality of Life for Indigenous and First Nations Peoples: A Mixed Methods Systematic Review
Source: Gerontologist. 2022 Oct 14;64(1):gnac153. doi: 10.1093/geront/gnac153 (PMC10733124; doi:10.1093/geront/gnac153)
Supplement: gnac153_suppl_Supplementary_Materials [file gnac153_suppl_supplementary_materials.docx]

**Online Supplementary Material**

**S1: Search Strategy**

CINAHL – Search ran on 24/08/2020

| Search no. | Search terms | Results |
| --- | --- | --- |
| S1 | (MM "Ethnic Groups") OR (MM "Cultural Diversity") OR (MM "Indigenous Peoples+") OR (MM "Minority Groups") | 33108 |
| S2 | TI ( ethnolinguistic OR "minority group*" OR ethnic* OR "minority race*" OR "ethnic minorit*" OR marginalis* OR indigenous OR "cultural* divers*" OR "linguistically diverse" OR "racial minorit*" OR "First Nation*") OR AB ( ethnolinguistic OR "minority group*" OR ethnic* OR "minority race*" OR "ethnic minorit*" OR marginalis* OR indigenous OR "cultural* divers*" OR "linguistically diverse" OR "racial minorit*" OR "First Nation*") | 76446 |
| S3 | S1 OR S2 | 94701 |
| S4 | (MM "Residential Facilities") OR (MM "Nursing Homes+") | 16996 |
| S5 | TI ( "Elderly care home*" OR eldercare OR "residential aged care facilit*" OR "aged care home" OR "long term residential care home*" OR "rest home*" OR "nursing home*" OR "long term care facilit*" OR "old age home*" ) OR AB ( "Elderly care home*" OR eldercare OR "residential aged care facilit*" OR "aged care home" OR "long term residential care home*" OR "rest home*" OR "nursing home*" OR "long term care facilit*" OR "old age home*" ) | 27231 |
| S6 | S4 OR S5 | 36221 |
| S7 | (MM "Cultural Safety") OR (MM "Gerontologic Care") OR (MM "Long Term Care") OR (MM "Health Services for the Aged") OR (MM "Health Services, Indigenous") | 36741 |
| S8 | TI ( services OR facilit* OR program* OR activit* OR "geriatric health services" OR "health services for the elderly" OR wellness OR "cross-cultural care" OR "cultural care" OR "culturally competent health care" OR "culturally congruent care" OR "old age assistance" OR "cultural* safe*" OR wellbeing OR "cultural* aware*" OR "cultural differen*" OR "cultural needs" OR "cultural sensitiv*" OR power OR racism OR dominan* OR neo-colonial* OR "social determinants of health" OR "social inclusion" OR "social exclusion" OR "social justice" OR ageism OR intersectionality ) OR AB ( services OR facilit* OR program* OR activit* OR "geriatric health services" OR "health services for the elderly" OR wellness OR "cross-cultural care" OR "cultural care" OR "culturally competent health care" OR "culturally congruent care" OR "old age assistance" OR "cultural* safe*" OR wellbeing OR "cultural* aware*" OR "cultural differen*" OR "cultural needs" OR "cultural sensitiv*" OR power OR racism OR dominan* OR neo-colonial* OR "social determinants of health" OR "social inclusion" OR "social exclusion" OR "social justice" OR ageism OR intersectionality) | 1084696 |
| S9 | S7 OR S8 | 1108636 |
| S10 | S3 AND S6 AND S9 | 336 |

Medline (via EbscoHost) - Search ran on 24/08/2020

| Search no. | Search terms | Results |
| --- | --- | --- |
| S1 | (MM "Indigenous Peoples+") OR (MM "Minority Groups") OR (MM "Ethnic Groups+") OR (MM "Cultural Diversity") | 97136 |
| S2 | TI ( ethnolinguistic OR "minority group*" OR ethnic* OR "minority race*" OR "ethnic minorit*" OR marginalis* OR indigenous OR "cultural* divers*" OR "linguistically diverse" OR "racial minorit*" OR "First Nation*" ) OR AB ( ethnolinguistic OR "minority group*" OR ethnic* OR "minority race*" OR "ethnic minorit*" OR marginalis* OR indigenous OR "cultural* divers*" OR "linguistically diverse" OR "racial minorit*" OR "First Nation*" ) | 178530 |
| S3 | S1 OR S2 | 247196 |
| S4 | (MM "Nursing Homes+") OR (MM "Homes for the Aged") OR (MM "Assisted Living Facilities") OR (MM "Residential Facilities+") | 35746 |
| S5 | TI ( "elderly care home*" OR eldercare OR "residential aged care facilit*" OR "aged care home*" OR "long term residential care home*" OR "rest home" OR "nursing home*" OR "long term care facilit*" OR "old age home*" ) OR AB ( "elderly care home*" OR eldercare OR "residential aged care facilit*" OR "aged care home*" OR "long term residential care home*" OR "rest home" OR "nursing home*" OR "long term care facilit*" OR "old age home*" ) | 35337 |
| S6 | S4 OR S5 | 54367 |
| S7 | (MM "Health Services for the Aged") OR (MM "Health Services, Indigenous") OR (MM "Minority Health") OR (MM "Facilities and Services Utilization") OR (MM "Program Development") OR (MM "Health Resources") OR (MM "Culturally Competent Care") | 31995 |
| S8 | TI ( services OR facilit* OR program* OR activit* OR "geriatric health services" OR "health services for the elderly" OR "wellness program*" OR "cross-cultural care" OR "cultural care" OR "culturally competent health care" OR "culturally congruent care" OR "old age assistance" OR "cultural* safe*" OR wellbeing OR "cultural* aware*" OR "cultural differen*" OR "cultural needs" OR "cultural sensitiv*" OR power OR racism OR dominan* OR neo-colonial* OR "social determinants of health" OR "social inclusion" OR "social exclusion" OR "social justice" OR ageism OR intersectionality) OR AB ( services OR facilit* OR program* OR activit* OR "geriatric health services" OR "health services for the elderly" OR "wellness program*" OR "cross-cultural care" OR "cultural care" OR "culturally competent health care" OR "culturally congruent care" OR "old age assistance" OR "cultural* safe*" OR wellbeing OR "cultural* aware*" OR "cultural differen*" OR "cultural needs" OR "cultural sensitiv*" OR power OR racism OR dominan* OR neo-colonial* OR "social determinants of health" OR "social inclusion" OR "social exclusion" OR "social justice" OR ageism OR intersectionality ) | 5142326 |
| S9 | S7 OR S8 | 5158957 |
| S10 | S3 AND S6 AND S9 | 469 |

APA PsycInfo - Search ran on 24/08/2020

| Search no. | Search terms | Results |
| --- | --- | --- |
| S1 | (MM "Minority Groups" OR MM "Racial and Ethnic Groups") OR (MM "Indigenous Populations" OR MM "Alaska Natives" OR MM "American Indians" OR MM "Inuit" OR MM "Pacific Islanders") | 28108 |
| S2 | TI ( ethnolinguistic OR "minority group*" OR ethnic* OR "minority race*" OR "ethnic minorit*" OR marginalis* OR indigenous OR "cultural* divers*" OR "linguistically diverse" OR "racial minorit*" OR "First Nation*" ) OR AB ( ethnolinguistic OR "minority group*" OR ethnic* OR "minority race*" OR "ethnic minorit*" OR marginalis* OR indigenous OR "cultural* divers*" OR "linguistically diverse" OR "racial minorit*" OR "First Nation*" ) | 113976 |
| S3 | S1 OR S2 | 127270 |
| S4 | (MM "Nursing Homes") AND (MM "Residential Care Institutions" OR MM "Assisted Living") | 311 |
| S5 | TI ( "elderly care home*" OR eldercare OR "residential aged care facilit*" OR "aged care home*" OR "long term residential care home*" OR "rest home" OR "nursing home*" OR "long term care facilit*" OR "old age home*" ) OR AB ( "elderly care home*" OR eldercare OR "residential aged care facilit*" OR "aged care home*" OR "long term residential care home*" OR "rest home" OR "nursing home*" OR "long term care facilit*" OR "old age home*" ) | 13483 |
| S6 | S4 OR S5 | 13537 |
| S7 | (MM "Cultural Sensitivity") AND (MM "Elder Care" OR MM "Health Care Utilization") | 30 |
| S8 | TI ( services OR facilit* OR program* OR activit* OR "geriatric health services" OR "health services for the elderly" OR "wellness program*" OR "cross-cultural care" OR "cultural care" OR "culturally competent health care" OR "culturally congruent care" OR "old age assistance" OR "cultural* safe*" OR wellbeing OR "cultural* aware*" OR "cultural differen*" OR "cultural needs" OR "cultural sensitiv*" OR power OR racism OR dominan* OR neo-colonial* OR "social determinants of health" OR "social inclusion" OR "social exclusion" OR "social justice" OR ageism OR intersectionality) OR AB ( services OR facilit* OR program* OR activit* OR "geriatric health services" OR "health services for the elderly" OR "wellness program*" OR "cross-cultural care" OR "cultural care" OR "culturally competent health care" OR "culturally congruent care" OR "old age assistance" OR "cultural* safe*" OR wellbeing OR "cultural* aware*" OR "cultural differen*" OR "cultural needs" OR "cultural sensitiv*" OR power OR racism OR dominan* OR neo-colonial* OR "social determinants of health" OR "social inclusion" OR "social exclusion" OR "social justice" OR ageism OR intersectionality ) | 1288764 |
| S9 | S7 OR S8 | 1288773 |
| S10 | S3 AND S6 AND S9 | 188 |

Embase - Search ran on 24/08/2020

| Search no. | Search terms | Results |
| --- | --- | --- |
| S1 | 'indigenous people'/exp/mj OR 'minority group'/exp/mj OR 'ethnic group'/exp/mj OR 'cultural diversity'/exp/mj | 64418 |
| S2 | ethnolinguistic:ab,ti OR 'minority group*':ab,ti OR ethnic*:ab,ti OR 'minority race*':ab,ti OR 'ethnic minorit*':ab,ti OR marginalis*:ab,ti OR indigenous:ab,ti OR 'cultural* divers*':ab,ti OR 'linguistically diverse':ab,ti OR 'racial minorit*':ab,ti OR 'first nation*':ab,ti | 245881 |
| S3 | S1 OR S2 | 294135 |
| S4 | 'nursing home'/exp/mj OR 'residential care'/mj OR 'home for the aged'/exp/mj | 33256 |
| S5 | 'elderly care home*':ab,ti OR eldercare:ab,ti OR 'residential aged care facilit*':ab,ti OR 'aged care home*':ab,ti OR 'long term residential care home*':ab,ti OR 'rest home*':ab,ti OR 'nursing home*':ab,ti OR 'long term care facilit*':ab,ti OR 'old age home*':ab,ti | 46599 |
| S6 | S4 OR S5 | 63605 |
| S7 | 'elderly care'/mj OR 'geriatric care'/mj OR 'transcultural care'/mj OR 'indigenous health care'/exp/mj OR 'long term care'/mj | 53945 |
| S8 | services:ab,ti OR facilit*:ab,ti OR program*:ab,ti OR activit*:ab,ti OR 'geriatric health services':ab,ti OR 'health services for the elderly':ab,ti OR 'wellness program*':ab,ti OR 'cross-cultural care':ab,ti OR 'cultural care':ab,ti OR 'culturally competent health care':ab,ti OR 'culturally congruent care':ab,ti OR 'old age assistance':ab,ti OR 'cultural* safe*':ab,ti OR wellbeing:ab,ti OR 'cultural* aware*':ab,ti OR 'cultural differen*':ab,ti OR 'cultural needs':ab,ti OR 'cultural sensitiv*':ab,ti OR power:ab,ti OR racism:ab,ti OR dominan*:ab,ti OR 'neo colonial*':ab,ti OR 'social determinants of health':ab,ti OR 'social inclusion':ab,ti OR 'social exclusion':ab,ti OR 'social justice':ab,ti OR ageism:ab,ti OR intersectionality:ab,ti | 6432198 |
| S9 | S7 OR S8 | 6470027 |
| S10 | S3 AND S6 AND S9 | 536 |

Nursing and Allied Health – Search ran on 25/08/2020

| Search no. | Search terms | Results |
| --- | --- | --- |
| S1 | ((MJMESH.EXACT("Cultural Diversity") OR MJMESH.EXACT.EXPLODE("Ethnic Groups:N.01.224.317") OR MJMESH.EXACT("Minority Groups") OR MJMESH.EXACT.EXPLODE("Ethnic Groups:M.01.686.754") OR MJMESH.EXACT.EXPLODE("Indigenous Peoples")) | 9604 |
| S2 | ab((ethnolinguistic OR "minority group*" OR ethnic* OR "minority race*" OR "ethnic minorit*" OR marginalis* OR indigenous OR "cultural* divers*" OR "linguistically diverse" OR "racial minorit*" OR "First Nation*")) OR ti((ethnolinguistic OR "minority group*" OR ethnic* OR "minority race*" OR "ethnic minorit*" OR marginalis* OR indigenous OR "cultural* divers*" OR "linguistically diverse" OR "racial minorit*" OR "First Nation*"))) | 58690 |
| S3 | S1 OR S2 | 65238 |
| S4 | ((MJMESH.EXACT("Assisted Living Facilities") OR MJMESH.EXACT("Homes for the Aged") OR MJMESH.EXACT("Residential Facilities") OR MJMESH.EXACT("Nursing Homes")) | 2289 |
| S5 | ab(elderly care home* OR eldercare OR "residential aged care facilit*" OR "aged care home*" OR "long term residential care home*" OR "rest home*" OR "nursing home*" OR "long term care facilit*" OR "old age home*") OR ti(elderly care home* OR eldercare OR "residential aged care facilit*" OR "aged care home*" OR "long term residential care home*" OR "rest home*" OR "nursing home*" OR "long term care facilit*" OR "old age home*")) | 22817 |
| S6 | S4 OR S5 | 23664 |
| S7 | ((MJMESH.EXACT("Health Resources") OR MJMESH.EXACT("Facilities and Services Utilization") OR MJMESH.EXACT("Culturally Competent Care") OR MJMESH.EXACT("Health Services for the Aged") OR MJMESH.EXACT("Minority Health") OR MJMESH.EXACT("Program Development") OR MJMESH.EXACT("Health Services, Indigenous")) | 2803 |
| S8 | ab(services OR facilit* OR program* OR activit* OR "geriatric health services" OR "health services for the elderly" OR wellness OR "cross-cultural care" OR "cultural care" OR "culturally competent health care" OR "culturally congruent care" OR "old age assistance" OR "cultural* safe*" OR wellbeing OR "cultural* aware*" OR "cultural differen*" OR "cultural needs" OR "cultural sensitiv*" OR power OR racism OR dominan* OR neo-colonial* OR "social determinants of health" OR "social inclusion" OR "social exclusion" OR "social justice" OR ageism OR intersectionality) OR ti(services OR facilit* OR program* OR activit* OR "geriatric health services" OR "health services for the elderly" OR wellness OR "cross-cultural care" OR "cultural care" OR "culturally competent health care" OR "culturally congruent care" OR "old age assistance" OR "cultural* safe*" OR wellbeing OR "cultural* aware*" OR "cultural differen*" OR "cultural needs" OR "cultural sensitiv*" OR power OR racism OR dominan* OR neo-colonial* OR "social determinants of health" OR "social inclusion" OR "social exclusion" OR "social justice" OR ageism OR intersectionality) | 1248139 |
| S9 | S7 OR S8 | 1249427 |
| S10 | S3 AND S6 AND S9 | 233 |

Health & Medical Collection – Search run on 24/08/2020

| Search no. | Search terms | Results |
| --- | --- | --- |
| S1 | ((MJMESH.EXACT("Cultural Diversity") OR MJMESH.EXACT("Ethnic Groups") OR MJMESH.EXACT("Minority Groups") OR MJMESH.EXACT.EXPLODE("Indigenous Peoples")) | 4749 |
| S2 | ab((ethnolinguistic OR "minority group*" OR ethnic* OR "minority race*" OR "ethnic minorit*" OR marginalis* OR indigenous OR "cultural* divers*" OR "linguistically diverse" OR "racial minorit*" OR "First Nation*")) OR ti((ethnolinguistic OR "minority group*" OR ethnic* OR "minority race*" OR "ethnic minorit*" OR marginalis* OR indigenous OR "cultural* divers*" OR "linguistically diverse" OR "racial minorit*" OR "First Nation*"))) | 98873 |
| S3 | S1 OR S2 | 101145 |
| S4 | ((MJMESH.EXACT("Assisted Living Facilities") OR MJMESH.EXACT("Homes for the Aged") OR MJMESH.EXACT("Residential Facilities") OR MJMESH.EXACT("Nursing Homes")) | 2701 |
| S5 | ab(elderly care home* OR eldercare OR "residential aged care facilit*" OR "aged care home*" OR "long term residential care home*" OR "rest home*" OR "nursing home*" OR "long term care facilit*" OR "old age home*") OR ti(elderly care home* OR eldercare OR "residential aged care facilit*" OR "aged care home*" OR "long term residential care home*" OR "rest home*" OR "nursing home*" OR "long term care facilit*" OR "old age home*")) | 27599 |
| S6 | S4 OR S5 | 28586 |
| S7 | ((MJMESH.EXACT("Health Resources") OR MJMESH.EXACT("Facilities and Services Utilization") OR MJMESH.EXACT("Culturally Competent Care") OR MJMESH.EXACT("Health Services for the Aged") OR MJMESH.EXACT("Minority Health") OR MJMESH.EXACT("Program Development") OR MJMESH.EXACT("Health Services, Indigenous")) | 3586 |
| S8 | ab(services OR facilit* OR program* OR activit* OR "geriatric health services" OR "health services for the elderly" OR wellness OR "cross-cultural care" OR "cultural care" OR "culturally competent health care" OR "culturally congruent care" OR "old age assistance" OR "cultural* safe*" OR wellbeing OR "cultural* aware*" OR "cultural differen*" OR "cultural needs" OR "cultural sensitiv*" OR power OR racism OR dominan* OR neo-colonial* OR "social determinants of health" OR "social inclusion" OR "social exclusion" OR "social justice" OR ageism OR intersectionality) OR ti(services OR facilit* OR program* OR activit* OR "geriatric health services" OR "health services for the elderly" OR wellness OR "cross-cultural care" OR "cultural care" OR "culturally competent health care" OR "culturally congruent care" OR "old age assistance" OR "cultural* safe*" OR wellbeing OR "cultural* aware*" OR "cultural differen*" OR "cultural needs" OR "cultural sensitiv*" OR power OR racism OR dominan* OR neo-colonial* OR "social determinants of health" OR "social inclusion" OR "social exclusion" OR "social justice" OR ageism OR intersectionality) | 2244920 |
| S9 | S7 OR S8 | 2246471 |
| S10 | S3 AND S6 AND S9 | 257 |

**S2: Excluded studies**

Administration for Community Living. Native Americans and Older Americans Act Services: A Series on Racial and Ethnic Minorities. 2019. ***Reason for exclusion: Not the phenomena or outcomes of interest***

Anonymous. Safety risk visits to Indigenous aged care services: ANJ. Australian Nursing Journal. 2008;16(5):6–7. ***Reason for exclusion: Not models or elements of care***

Australian Institute of Health and Welfare. Insights into vulnerabilities of Aboriginal and Torres Strait Islander people aged 50 and over 2019. 2019. ***Reason for exclusion: Not models or elements of care***

Australian Institute of Health and Welfare. Towards better Indigenous health data. 2013. ***Reason for exclusion: Not the phenomena or outcomes of interest***

Australian National Audit Office. Indigenous Aged Care. 2017. ***Reason for exclusion: Not models or elements of care***

Blackford-Busson D. Residents’ perceptions of quality of life in a culturally diverse long-term care environment. 2013. ***Reason for exclusion: Not Indigenous population***

Bowblis JR, Ng W, Akosionu O, Shippee TP. Decomposing Racial and Ethnic Disparities in Nursing Home Quality of Life. Journal of Applied Gerontology. 2020. ***Reason for exclusion: The nursing home with Native American population was excluded from the data analysis***

Boyd-Seale DL. Cultural competency in nursing homes’ activities programs. 2008. ***Reason for exclusion: No outcomes on quality of life or consumer satisfaction***

Broe T. What do Aboriginal Australians want from their aged care system? Community connection is number one. 2019. ***Reason for exclusion: Opinion piece with no references***

Brooke NJ. Needs of Aboriginal and Torres Strait Islander clients residing in Australian residential aged‐care facilities. The Australian Journal of Rural Health. 2011;19(4):166–70. ***Reason for exclusion: Systematic review paper***

Browne C, Kaopua LS. Dementia in native populations; making the case for culturally tailored dementia care with indigenous elders and families. Neurodegenerative Diseases. 2015;15:1051. ***Reason for exclusion: Not the phenomena or outcomes of interest***

DHSS Victoria. Korin Korin Balit-Djak Aboriginal health, wellbeing and safety strategic plan 2017–2027. 2017. ***Reason for exclusion: Not the phenomena or outcomes of interest***

Dance P, Brown R, Bammer G, Sibthorpe B. Aged care services for Indigenous people in the Australian Capital Territory and surrounds:analysing needs and implementing change. Australian and New Zealand journal of public health. 2004;28(6):579–83. ***Reason for exclusion: Not the phenomena or outcomes of interest***

Davy C, Kite E, Aitken G, Dodd G, Rigney J, Hayes J, et al. What keeps you strong? A systematic review identifying how primary health‐care and aged‐care services can support the well‐being of older Indigenous peoples. Australasian journal on ageing. 2016;35(2):90–7. ***Reason for exclusion: Systematic review paper***

Department of Health. Shared actions to support all diverse older people: A guide for aged care providers. 2019. ***Reason for exclusion: Not Indigenous population***

Department of Health. WA Aboriginal Health and Wellbeing Framework 2015–2030. 2015. ***Reason for exclusion: Not models or elements of care***

Donna MG, Donald W, Valerie E. The Impact of History and Culture on Nursing Care of Native American Elders. Journal of gerontological nursing. 2012;38(10):3–5. ***Reason for exclusion: Not the phenomena or outcomes of interest***

Douglas D, Traynor V, Burns P. INDIGENOUS AUNTIES: DEMENTIA AND DANCE. Australian Nursing & Midwifery Journal. 2018;25(7):40. ***Reason for exclusion: Not models or elements of care***

ELDAC. Aboriginal and Torres Strait Islander Peoples - Information and Services - Population Groups. 2020. ***Reason for exclusion: Focus on palliative care from review of literature***

Hobus RM. Living in two worlds: a Lakota transcultural nursing experience. Journal of transcultural nursing : official journal of the Transcultural Nursing Society / Transcultural Nursing Society. 1990;2(1):33–6. ***Reason for exclusion: Not the phenomena or outcomes of interest***

Hocking B, Lowe M, Nagel T, Phillips C, Lindeman M, Farthing A, et al. Dementia in Aboriginal people in Residential Aged Care Facilities in Alice Springs: A Descriptive Study. Brain Impairment. 2019;20(2):171–9. ***Reason for exclusion: Not the phenomena or outcomes of interest***

Jervis LL, Manson SM. Cognitive impairment, psychiatric disorders, and problematic behaviors in a tribal nursing home. Journal of Aging & Health. 2007;19(2):260–74. ***Reason for exclusion: Not the outcomes of interest.***

K. Smith, J. J. Grundy, H. J. Nelson. Culture at the centre of community based aged care in a remote Australian Indigenous setting: a case study of the development of Yuendumu Old People’s Programme. Rural and remote health. 2010;10(4):1422. ***Reason for exclusion: Not residential or long term or nursing home care***

Lyman AJ, Edwards ME. Poetry: Life review for frail American Indian elderly. Journal of Gerontological Social Work. 1989;14(1–2):75–91. ***Reason for exclusion: Not outcomes of interest***

MacLean MJ, Sakadakis V. Quality of life in terminal care with institutionalized ethnic elderly people. International Social Work. 1989;32(3):209–21. ***Reason for exclusion: Not Indigenous population***

Manson SM. Provider assumptions about long-term care in American Indian communities. The Gerontologist. 1989;29(3):355–8. ***Reason for exclusion: Not the phenomena or outcomes of interest***

Mazanec P, Tyler MK. Cultural considerations in end-of-life care: how ethnicity, age, and spirituality affect decisions when death is imminent...originally published in AJN, the American Journal of Nursing, 103(3), 50-58. Reprinted with permission. Home Healthcare Nurse. 2004;22(5):317–26. ***Reason for exclusion: Not Indigenous populations***

Ministry of Health New Zealand. The Health and Wellbeing of Older People and Kaumätua: The Public Health Issues. 1997. **R*eason for exclusion: Not the phenomena or outcomes of interest***

Mold F, Fitzpatrick JM, Roberts JD. Care of older people. Caring for minority ethnic older people in nursing care homes. British Journal of Nursing. 2005;14(11):601–6. ***Reason for exclusion: Not Indigenous populations***

Mold F, Fitzpatrick JM, Roberts JD. Minority ethnic elders in care homes: A review of the literature. Age and Ageing. 2005;34(2):107–13. ***Reason for exclusion: Not Indigenous population***

Montayre J, Montayre J, Thaggard S. Culturally and Linguistically Diverse Older Adults and Mainstream Long-Term Care Facilities: Integrative Review of Views and Experiences. Research in Gerontological Nursing. 2018;11(5):265–76. ***Reason for exclusion: Not Indigenous population***

Mowbray M. Social determinants and Indigenous health: The International experience and its policy implications. Report on specially prepared documents, presentations and discussion at the International Symposium on the Social Determinants of Indigenous Health Adelaide, 29-30 April 2007 for the Commission on Social Determinants of Health (CSDH). 2007. ***Reason for exclusion: Not the phenomena or outcomes of interest***

New Zealand Aged Care Association. Health of Older People Strategy 2016–2026: Consultation submissions. 2016. ***Reason for exclusion: Not Indigenous population***

Plotnikoff GA, Numrich C, Yang D, Chu Yongyuan W, Xiong P. Shamans and Conventional Care: Are We Prepared? HEC Forum. 2002;14(3):271–8. ***Reason for exclusion: Not Indigenous population, residential care or phenomena of interest***

Queensland Health. Aboriginal and Torres Strait islander Cultural Capability Framework 2010-2033. 2010. ***Reason for exclusion: Not the phenomena or outcomes of interest***

Radford K AW, Anderson M TA. Sharing the Wisdom of Our Elders Final Report. 2019. ***Reason for exclusion: Not models or elements of care***

Radford K, Mack H, Robertson H, Draper B, Chalkley S, Daylight G, et al. The Koori Growing Old Well Study: investigating aging and dementia in urban Aboriginal Australians. International Psychogeriatrics. 2014;26(6):1033–1043. ***Reason for exclusion: Not the phenomena or outcomes of interest***

Roscelli M. Political advocacy and research both needed to address federal-provincial gaps in service: Manitoba First Nations Personal Care Homes. Canadian journal of public health = Revue canadienne de sante publique. 2005;96 Suppl 1:S55–9. ***Reason for exclusion: Background paper. Not the phenomena of interest***

Shahid S, Taylor EV, Cheetham S, Woods JA, Aoun SM, Thompson SC. Key features of palliative care service delivery to Indigenous peoples in Australia, New Zealand, Canada and the United States: a comprehensive review. BMC palliative care. 2018;17(1):72. ***Reason for exclusion: Not residential or long term or nursing home care***

The University of Auckland. Intervals of care need: need for care and support in advanced age. 2011. ***Reason for exclusion: Not the phenomena or outcomes of interest***

Thurecht K. The Best of Aboriginal and Torres Strait Islander Aged Care. Aboriginal and islander health worker journal. 2010;34(6):32–3. ***Reason for exclusion: Opinion piece***

United Nations. State of the Worlds Indigenous Peoples: Indigenous Peoples’ Access to Health Services. 2015. ***Reason for exclusion: Not the phenomena or outcomes of interest***

University of New South Wales. Growing Old Well: A Life Cycle Approach for Aboriginal and Torres Strait Islander People. 2008. ***Reason for exclusion: Not models or elements of care***

Xiao LD, Willis E, Harrington A, Gillham D, De Bellis A, Morey W, et al. Improving socially constructed cross-cultural communication in aged care homes: A critical perspective. Nursing inquiry. 2018;25(1). ***Reason for exclusion: Not Indigenous population***

du Toit SHJ, Baldassar L, Raber CL, Millard AM, Etherton-Beer CD, Buchanan HA, et al. Embracing Cultural Diversity - Leadership Perspectives on Championing Meaningful Engagement for Residents Living with Advanced Dementia. Journal of Cross-Cultural Gerontology. 2020;35(1):49–67. ***Reason for exclusion: Not Indigenous population***

**S3: PRISMA Checklist**

| **Section and Topic** | **Item #** | **Checklist item** | **Location where item is reported** |
| --- | --- | --- | --- |
| **TITLE** | | |  |
| Title | 1 | Identify the report as a systematic review. | Title page |
| **ABSTRACT** | | |  |
| Abstract | 2 | See the PRISMA 2020 for Abstracts checklist. | Abstract |
| **INTRODUCTION** | | |  |
| Rationale | 3 | Describe the rationale for the review in the context of existing knowledge. | Background and objectives p4-6 |
| Objectives | 4 | Provide an explicit statement of the objective(s) or question(s) the review addresses. | Background and objectives p6 |
| **METHODS** | | |  |
| Eligibility criteria | 5 | Specify the inclusion and exclusion criteria for the review and how studies were grouped for the syntheses. | Eligibility criteria p6-7 |
| Information sources | 6 | Specify all databases, registers, websites, organisations, reference lists and other sources searched or consulted to identify studies. Specify the date when each source was last searched or consulted. | Search strategy p7-8 Supplementary material S1 |
| Search strategy | 7 | Present the full search strategies for all databases, registers and websites, including any filters and limits used. | Supplementary material S1 |
| Selection process | 8 | Specify the methods used to decide whether a study met the inclusion criteria of the review, including how many reviewers screened each record and each report retrieved, whether they worked independently, and if applicable, details of automation tools used in the process. | Selection and critical appraisal p8 |
| Data collection process | 9 | Specify the methods used to collect data from reports, including how many reviewers collected data from each report, whether they worked independently, any processes for obtaining or confirming data from study investigators, and if applicable, details of automation tools used in the process. | Data extraction p8-9 |
| Data items | 10a | List and define all outcomes for which data were sought. Specify whether all results that were compatible with each outcome domain in each study were sought (e.g. for all measures, time points, analyses), and if not, the methods used to decide which results to collect. | Data extraction p8-9 |
|  | 10b | List and define all other variables for which data were sought (e.g. participant and intervention characteristics, funding sources). Describe any assumptions made about any missing or unclear information. | Data extraction p7-8 |
| Study risk of bias assessment | 11 | Specify the methods used to assess risk of bias in the included studies, including details of the tool(s) used, how many reviewers assessed each study and whether they worked independently, and if applicable, details of automation tools used in the process. | Selection and critical appraisal p8 |
| Effect measures | 12 | Specify for each outcome the effect measure(s) (e.g. risk ratio, mean difference) used in the synthesis or presentation of results. | Not applicable as qualitative review |
| Synthesis methods | 13a | Describe the processes used to decide which studies were eligible for each synthesis (e.g. tabulating the study intervention characteristics and comparing against the planned groups for each synthesis (item #5)). | Data extraction and synthesis p8-9 |
|  | 13b | Describe any methods required to prepare the data for presentation or synthesis, such as handling of missing summary statistics, or data conversions. | Data extraction and synthesis p8-9 |
|  | 13c | Describe any methods used to tabulate or visually display results of individual studies and syntheses. | Data extraction and synthesis p8-9 |
|  | 13d | Describe any methods used to synthesize results and provide a rationale for the choice(s). If meta-analysis was performed, describe the model(s), method(s) to identify the presence and extent of statistical heterogeneity, and software package(s) used. | Data extraction and synthesis p8-9 |
|  | 13e | Describe any methods used to explore possible causes of heterogeneity among study results (e.g. subgroup analysis, meta-regression). | Data extraction and synthesis p8-9 |
|  | 13f | Describe any sensitivity analyses conducted to assess robustness of the synthesized results. | N/A |
| Reporting bias assessment | 14 | Describe any methods used to assess risk of bias due to missing results in a synthesis (arising from reporting biases). | Selection and critical appraisal p8 |
| Certainty assessment | 15 | Describe any methods used to assess certainty (or confidence) in the body of evidence for an outcome. | Selection and critical appraisal p8 |
| **RESULTS** | | |  |
| Study selection | 16a | Describe the results of the search and selection process, from the number of records identified in the search to the number of studies included in the review, ideally using a flow diagram. | Results p9-10  PRISMA flow diagram (Figure 1). |
|  | 16b | Cite studies that might appear to meet the inclusion criteria, but which were excluded, and explain why they were excluded. | Results p9  Supplementary material S2 |
| Study characteristics | 17 | Cite each included study and present its characteristics. | Results p9-11  Tables 1 and 2 |
| Risk of bias in studies | 18 | Present assessments of risk of bias for each included study. | Results p11  Supplementary material S4 |
| Results of individual studies | 19 | For all outcomes, present, for each study: (a) summary statistics for each group (where appropriate) and (b) an effect estimate and its precision (e.g. confidence/credible interval), ideally using structured tables or plots. | N/A |
| Results of syntheses | 20a | For each synthesis, briefly summarise the characteristics and risk of bias among contributing studies. | Results p9-16  Table 3  Supplementary material S5 |
|  | 20b | Present results of all statistical syntheses conducted. If meta-analysis was done, present for each the summary estimate and its precision (e.g. confidence/credible interval) and measures of statistical heterogeneity. If comparing groups, describe the direction of the effect. | N/A |
|  | 20c | Present results of all investigations of possible causes of heterogeneity among study results. | N/A |
|  | 20d | Present results of all sensitivity analyses conducted to assess the robustness of the synthesized results. | N/A |
| Reporting biases | 21 | Present assessments of risk of bias due to missing results (arising from reporting biases) for each synthesis assessed. | N/A |
| Certainty of evidence | 22 | Present assessments of certainty (or confidence) in the body of evidence for each outcome assessed. | Conqual summary of findings p16  Supplementary material S5 |
| **DISCUSSION** | | |  |
| Discussion | 23a | Provide a general interpretation of the results in the context of other evidence. | Discussion p16-19 |
|  | 23b | Discuss any limitations of the evidence included in the review. | Limitations p19 |
|  | 23c | Discuss any limitations of the review processes used. | Limitations p19 |
|  | 23d | Discuss implications of the results for practice, policy, and future research. | Implications for practice p20 |
| **OTHER INFORMATION** | | |  |
| Registration and protocol | 24a | Provide registration information for the review, including register name and registration number, or state that the review was not registered. | Objectives p5-6 |
|  | 24b | Indicate where the review protocol can be accessed, or state that a protocol was not prepared. | Objectives p5-6 |
|  | 24c | Describe and explain any amendments to information provided at registration or in the protocol. | N/A |
| Support | 25 | Describe sources of financial or non-financial support for the review, and the role of the funders or sponsors in the review. | Funding p2 |
| Competing interests | 26 | Declare any competing interests of review authors. | Conflict of Interest p2 |
| Availability of data, code and other materials | 27 | Report which of the following are publicly available and where they can be found: template data collection forms; data extracted from included studies; data used for all analyses; analytic code; any other materials used in the review. | Supplementary material supplied and referenced in text |

*From:*  Page MJ, McKenzie JE, Bossuyt PM, Boutron I, Hoffmann TC, Mulrow CD, et al. The PRISMA 2020 statement: an updated guideline for reporting systematic reviews. BMJ 2021;372:n71. doi: 10.1136/bmj.n71

For more information, visit: <http://www.prisma-statement.org/>

**S4: Assessment of Methodological Quality of Qualitative Studies**

| **Citation** | **Q1** | **Q2** | **Q3** | **Q4** | **Q5** | **Q6** | **Q7** | **Q8** | **Q9** | **Q10** |
| --- | --- | --- | --- | --- | --- | --- | --- | --- | --- | --- |
| Brown and Gibbons (2008) | N | Y | U | U | U | N | N | N | Y | U |
| Du Toit et al. (2014) | Y | Y | Y | Y | U | U | N | U | U | Y |
| Hanssen and Kuven (2016) | Y | Y | Y | Y | Y | N | N | Y | Y | Y |
| Hendrix (2003) | U | U | U | U | U | U | Y | U | U | N |
| Kataoka-Yahiro et al. (2016) | U | Y | Y | Y | Y | N | N | Y | Y | Y |
| Mercer (1996) | Y | Y | Y | Y | Y | N | Y | Y | N | Y |
| Schultz and Helander (1988) | U | U | Y | Y | Y | N | N | N | N | Y |
| Selle (2007) | U | Y | Y | Y | Y | N | U | Y | U | Y |
| Shomaker (1981) | U | U | U | U | U | N | N | N | N | U |
| Sivertsen et al. (2019) | Y | Y | Y | Y | Y | Y | N | Y | Y | Y |
| Swinton (2011) | Y | Y | Y | Y | Y | N | N | Y | U | Y |
| % | 45.45 | 72.72 | 72.72 | 72.72 | 63.63 | 9.09 | 18.18 | 54.54 | 36.36 | 72.72 |

Q1) congruity between the stated philosophical perspective and the research methodology; Q2) congruity between the research methodology and the research question or objectives; Q3) congruity between the research methodology and the data collection methods; Q4) congruity between the research methodology and the representation and analysis of data; Q5) congruity between the research methodology and the interpretation of results; Q6) if there was a statement locating the researcher culturally or theoretically; Q7) whether the influence of the researcher on the research process and interpretations was addressed; Q8) if there was adequate representation of participants and their voices; Q9) if there was evidence of ethical approval for the research by an appropriate body; Q10) whether conclusions drawn appeared to flow from the analysis or interpretation of the data.

Y=yes; N=no; U=unclear

**S4: Assessment of Methodological Quality of Textual Reports**

| **Citation** | **Q1** | **Q2** | **Q3** | **Q4** | **Q5** | **Q6** |
| --- | --- | --- | --- | --- | --- | --- |
| Australian Government DoH (2019a) | Y | Y | Y | Y | N | N/A |
| Australian Government DoH (2019b) | Y | Y | Y | U | N | N/A |
| Health Quality & Safety Commission New Zealand (2019) | Y | Y | Y | U | N | N/A |
| National Aboriginal Community Controlled Health Organisation [NACCHO] (2019) | Y | Y | Y | Y | Y | N/A |
| National Advisory Group for Aboriginal and Torres Strait Islander Aged Care [NAGATSIAC] (2019) | Y | Y | Y | U | Y | Y |
| Office of Evaluation and Audit [OEA] (2009) | Y | Y | Y | Y | Y | N/A |
| Regional Development Council of Western Australia [RDCWA] (2016) | Y | U | Y | Y | N | N/A |
| % | 100.0 | 85.71 | 100.0 | 57.14 | 42.85 | 14.28 |

Q1) whether the source of opinion had been clearly identified; Q2) whether the source of opinion had standing in the field of expertise; Q3) whether the interests of the residents were the central focus of the opinion; Q4) Whether the opinions basis in logic or experience were clearly argued; Q5) whether there was reference to the extant literature or evidence that is non-biased and inclusive; Q6) whether the opinion was supported by peers within the available published literature.

Y=yes; N=no; U=unclear

**S5: ConQual Summary Findings**

| Synthesised finding | Papers contributing to finding | Dependability | Credibility | ConQual score | Explanation of assessment |
| --- | --- | --- | --- | --- | --- |
| LTC services should be co-designed and provided in collaboration with Indigenous and First Nations communities and organisations to help ensure culturally safe care and to recruit and retain local Indigenous workers | DoHa  DoHb  Du Toit  Mercer  NACCHO  NAGATSIAC  OEA  RDCWA  Shomaker  Sivertsen  Swinton | Very low to moderate | High | Moderate confidence in findings | Methodological quality was rated as very low to moderate; credibility of findings was rated as unequivocal |
| LTC facilities need to embed trauma informed care policies, practices and staff training to deliver culturally safe services to members of the Stolen Generation | DoHa  DoHb  Du Toit  NACCHO  NAGATSIAC  RDCWA  Sivertsen  Swinton | Low to moderate | High | Moderate confidence in findings | Methodological quality was rated as low to moderate; credibility of findings was rated as unequivocal |
| LTC facilities should meet the individual needs of Indigenous and First Nations residents in a culturally respectful way that includes upholding cultural, spiritual and religious beliefs, activities and practices, including those surrounding end of life | DoHa  DoHb  Du Toit  Hanssen  Hendrix  HQSC  Mercer  NAGATSIAC  NACCHO  OEA  RDCWA  Selle  Schultz  Shomaker  Sivertsen  Swinton | Very low to moderate | Medium to High | Moderate confidence in findings | Methodological quality was rated as very low to moderate; credibility of findings was rated as credible to unequivocal |
| Culturally safe LTC includes the sustained involvement of family, kin, and the wider Indigenous community to maintain connection to culture and sense of belonging | Brown  DoHa  DoHb  Du Toit  HQSC  Katoaka-Yahiro  Mercer  NAGATSIAC  OEA  Schultz  Shomaker  Swinton | Very low to moderate | Medium to High | Moderate confidence in findings | Methodological quality was rated as very low to moderate; credibility of findings was rated as credible to unequivocal |
